# Evolutionary dynamics of multidrug resistant *Salmonella enterica* serovar 4,[5],12:i:- in Australia

**DOI:** 10.1038/s41467-021-25073-w

**Published:** 2021-08-09

**Authors:** Danielle J. Ingle, Rebecca L. Ambrose, Sarah L. Baines, Sebastian Duchene, Anders Gonçalves da Silva, Darren Y. J. Lee, Miriam Jones, Mary Valcanis, George Taiaroa, Susan A. Ballard, Martyn D. Kirk, Benjamin P. Howden, Jaclyn S. Pearson, Deborah A. Williamson

**Affiliations:** 1 Research School of Population Health, Australian National University, Canberra, ACT Australia; 2 Microbiological Diagnostic Unit Public Health Laboratory, Department of Microbiology and Immunology, The University of Melbourne at The Peter Doherty Institute for Infection and Immunity, Melbourne, VIC Australia; 3 Department of Microbiology and Immunology, The University of Melbourne at The Peter Doherty Institute for Infection and Immunity, Melbourne, VIC Australia; 4 Centre for Innate Immunity and Infectious Diseases, Hudson Institute of Medical Research, Melbourne, VIC Australia; 5 Department of Molecular and Translational Research, Monash University, Melbourne, VIC Australia; 6 Department of Microbiology, Monash University, Melbourne, VIC Australia; 7 Department of Microbiology, Royal Melbourne Hospital, Melbourne, VIC Australia

**Keywords:** Phylogenetics, Bacteriology, Bacterial genetics

## Abstract

*Salmonella enterica* serovar 4,[5],12:i:- (*Salmonella* 4,[5],12:i:-) is a monophasic variant of *Salmonella* Typhimurium that has emerged as a global cause of multidrug resistant salmonellosis. We used Bayesian phylodynamics, genomic epidemiology, and phenotypic characterization to describe the emergence and evolution of *Salmonella* 4,[5],12:i:- in Australia. We show that the interruption of the genetic region surrounding the phase II flagellin, FljB, causing a monophasic phenotype, represents a stepwise evolutionary event through the accumulation of mobile resistance elements with minimal impairment to bacterial fitness. We identify three lineages with different population dynamics and discrete antimicrobial resistance profiles emerged, likely reflecting differential antimicrobial selection pressures. Two lineages are associated with travel to South-East Asia and the third lineage is endemic to Australia. Moreover antimicrobial-resistant *Salmonella* 4,[5],12:i- lineages efficiently infected and survived in host phagocytes and epithelial cells without eliciting significant cellular cytotoxicity, suggesting a suppression of host immune response that may facilitate the persistence of *Salmonella* 4,[5],12:i:-.

## Abstract (web summary)

*Salmonella enterica* serovar 4,[5],12:i:- (*Salmonella* 4,[5],12:i:-) is a major pathogen of humans and animals with a reported incidence in Australia three times higher than the UK and USA. Here, the authors report the circulation, antimicrobial resistance signatures, and effects on host cells, of three *Salmonella*4,[5],12:i:- lineages within Australia.

## Introduction

Non-typhoidal *Salmonella enterica* (NTS) is a major pathogen of humans and animals, and is responsible for approximately 153 million cases of gastroenteritis and 57,000 deaths globally each year^1^. Unlike human-restricted typhoidal *Salmonella* (serovars Typhi and Paratyphi A, B, and C), one of the key features of non-typhoidal *Salmonella* (NTS) is a wide host range^2^. In particular, *S*. *enterica* serovar Typhimurium (*S*. Typhimurium) is considered the prototype host generalist, with a broad zoonotic and environmental reservoir^3–5^. *S*. Typhimurium is a leading cause of human salmonellosis, with a key epidemiological feature over the past few decades being sequential clonal expansions of multidrug-resistant (MDR) *S*. Typhimurium lineages, including phage type DT204, DT29, and DT104^2,4,6,7^. *S*. Typhimurium lineages are characterized by several multi-locus sequence types (STs), including ST19, ST34, ST36, and ST313, and monophasic variants of significant public health concern have been detected in ST34 and ST313^8–10^.

Most recently, *Salmonella enterica* serovar 4,[5],12:i:- (*Salmonella* 4,[5],12:i:-), a monophasic variant of *S*. Typhimurium belonging to ST34, has emerged as a major global cause of NTS disease in animals and humans^11–17^. Key features of this pandemic lineage initially referred to as the European Clone due to its suggested origins^18^, are (i) deletion(s) in the phase II flagellin locus preventing the expression of the second flagella antigen FljB^8^, (ii) genes associated with heavy metal resistance, and (iii) increasingly extensive MDR^6,13,14,19^. The antimicrobial resistance (AMR) profile of resistance to ampicillin, streptomycin, sulfonamides, and tetracycline (ASSuT) initially acted as an epidemiological marker for this lineage^16,20,21^. Of recent concern, however, are reports of resistance to third-generation cephalosporins and colistin in *Salmonella* 4,[5],12:i:- from Europe, North America, and parts of Asia^11,22,23^, raising the prospect of increasingly limited antimicrobial therapeutic options in cases of severe human salmonellosis^24^.

In addition to bacterial traits, the host range of *Salmonella* 4,[5],12:i:- also plays a role in the success of this global pathogen, with outbreaks linked to food products derived from livestock^11,12,25,26^, particularly swine. For example, colistin resistance has been reported in *Salmonella* 4,[5],12:i:- isolates from pigs and humans in China, where there has been use of polymyxins as growth supplements in swine^14,18,27^. Further, *mcr* genes mediating colistin resistance have also been identified on plasmids with resistance genes to multiple antimicrobials, including third-generation cephalosporins (3GCs)^14,18,23^. IncHI2 plasmids encoding both *mcr-3* and *bla*_CTX-M-55_ have been linked to clinical disease caused by *Salmonella* 4,[5],12:i:- in south-east Asia^14,18,23^.

In Australia, the reported incidence of human salmonellosis is more than three times higher than in the United Kingdom and the United States. Over the past 5 years, *Salmonella* 4,[5],12:i:- has emerged as one of the major serovars responsible for salmonellosis in Australia, and the most common MDR NTS^6,28^. To date, however, the specific factors contributing to the emergence of *Salmonella* 4,[5],12:i:- in Australia have not been assessed. Accordingly, to investigate the origins and establishment of *Salmonella* 4,[5],12:i:-, we undertook a combined genomic and phenotypic analysis, contextualizing Australian data with *Salmonella* 4,[5],12:i:- isolates from Europe, Asia, and the Americas. We show the circulation of three major global lineages of *Salmonella* 4,[5],12:i:- within Australia, each associated with different AMR profiles to key antimicrobials and signature deletions in the phase II flagellin locus. Further, we demonstrate that some *Salmonella* 4,[5],12:i:- exhibit an increased ability to replicate in host cells without eliciting increased cellular cytotoxicity, suggesting an enhanced capacity for immune evasion and potentially providing a competitive advantage over other NTS.

## Results

### Global phylogeography of *Salmonella* 4,[5],12:i:- and multiple introductions into Australia

**Fig. 1:**
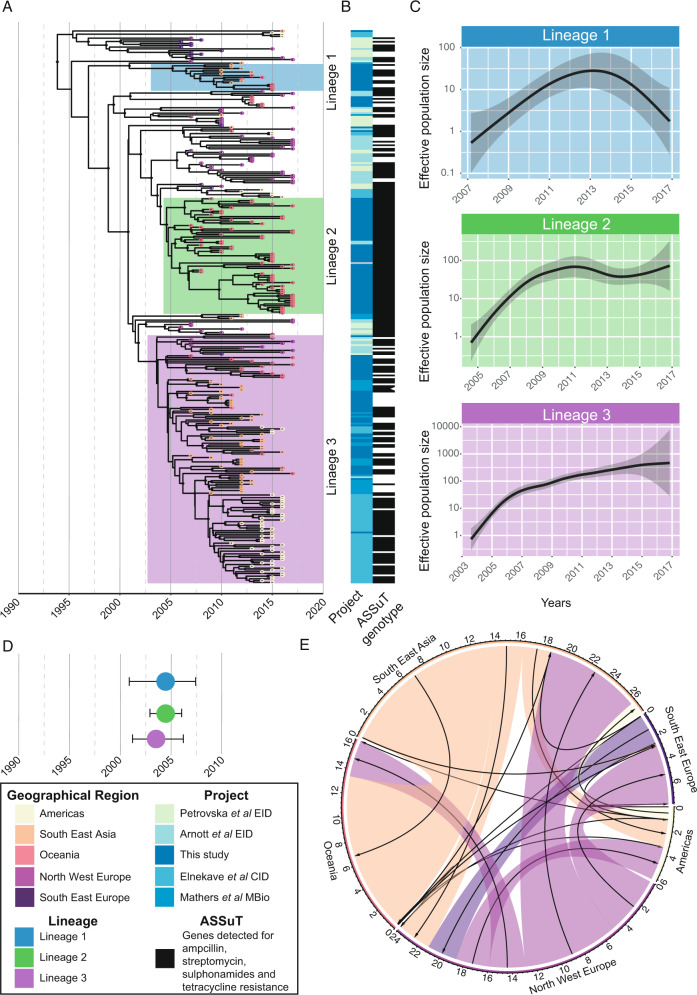
Inferred population dynamics of ST34 *Salmonella* 4,[5],12:i:-. **A** Bayesian evolutionary analysis showing the maximum-clade credibility (MCC) tree of ST34 isolates (*n* = 309) inferred from 2693 SNPs, demonstrating the timing of divergence and geographical spread of *Salmonella* 4,[5],12:i:- globally. The tips of the phylogeny are colored by the major geographical regions for the reported country of collection (for publicly available data) or by destination of reported travel (if any) for Australian isolates. The three lineages are highlighted on the phylogeny. The posterior support of ≥0.95 for internal nodes is shown with black circles on the node. The time in years is given on the *x* axis. Phylogeny available in Newick form from microreact (https://microreact.org/project/mfxxBchBsUpsJu7nvfkFw4). **B** Heatmap that shows the study from which the isolates were sourced and the presence of the ASSuT genotype (defined by any genes mediating antimicrobial resistance to ampicillin, streptomycin, sulfonamides, and tetracycline). **C** The estimated effective population size through time for each of the three lineages. The shaded area indicates the 95% confidence interval. **D** Visualization of the most recent common ancestor (MRCA) of the three lineages with highest posterior density (HPD) intervals shown as the bars. **E** Circular migration diagram of the migration events between the five geographical regions. The size of the colored block denotes the posterior mean number of inferred migration events from the Bayesian phylogeographical analysis and arrows denote directionality. The inset box is the legend for the different attributes.

To investigate the introduction of *Salmonella* 4,[5],12:i:- into Australia and define the geographic distribution of lineages, we undertook phylogenomic analyses of 136 Australian isolates, contextualized with 173 publicly available isolates from multiple geographical regions (Fig. 1A, B and Supplementary Fig. 1A, B, Supplementary Data 1)^8,11,12,19^. Bayesian phylodynamic analyses from the 2693 core SNP alignment, using the highest supported model of a relaxed clock and coalescent exponential tree prior, revealed three major circulating lineages. These lineages were defined by the most recent common ancestor (MRCA) of the clusters of >10 Australian isolates (Fig. 1A) and had high posterior probability support of ≥0.95 (shown in the maximum-clade-credibility [MCC] tree in Fig. 1A).

One of these lineages, Lineage 2, contained isolates mainly from cases in Australia with no reported overseas travel. Further, Lineage 2 also contained six ST34 *Salmonella* 4,[5],12:i:- isolated from cattle in Australia and a previously published swine isolate from Australia^29^. In contrast, Australian isolates in Lineages 1 and 3 were associated with cases that reported recent international travel to South-East Asia. Similar phylogeographic associations were observed for isolates from the United States, which clustered together and formed part of an endemic lineage prevalent in pigs in the United States, as previously reported^11^. In our phylogeographic analysis, these United States isolates were most closely related to isolates collected from pigs in Vietnam (Fig. 1A)^12^.

Differences in the effective population size (*Ne*) trajectories, a measure of genetic diversity, were observed between Australian isolates belonging to the three lineages (Fig. 1C), suggesting Lineage 2 and Lineage 3 both underwent population expansion since their emergence in the early 2000s, which has stabilized in recent years. In contrast, the *Ne* for Lineage 1 suggested a population that initially increased, with a subsequent decrease over the past five years (Fig. 1C).

We assessed for temporal signal within the dataset (Supplementary Fig 1C). To test the robustness of the molecular clock signal, we employed a Bayesian evaluation of temporal signal (BETS)^30^. A strong temporal signal within ST34 *Salmonella* 4,[5],12:i:- was shown for the model with correct sampling times with a log Bayes factor of 246 relative to the same model with no sampling times. The substitution rate (the number of expected substitutions per site per year) was estimated at 3.88E−7 (95% highest posterior density [HPD] 3.28E−7 to 4.46E−7) corresponding to previous estimates for non-typhoidal *S. enterica* serovars^31^. The year of emergence of the MRCA for all included ST34 *Salmonella* 4,[5],12:i:- was estimated at 1992 (HPD 1987–1996), with likely concurrent circulation of all three lineages in Australia since the turn of the twenty-first century (Fig. 1D).

We then used a mugration model (which uses stochastic mapping to infer ancestral states) to infer ancestral geographic locations^32^. We included travel history data, where available for Australian isolates^33^. The posterior support for the geographical location provided strong evidence supporting the emergence of ST34 *Salmonella* 4,[5],12:i:- in the northern hemisphere, with subsequent spread to South-East Asia and Australia at the turn of the twenty-first century (Fig. 1A and Supplementary Fig. 2A). We also explored the migration dynamics of ST34 *Salmonella* 4,[5],12:i:- using a phylogeographic approach that estimated the putative number of migration events between our five included geographical regions^33^. Most migration events for Oceania were importation events with a total of 13.2 importations [HPD 9-17] from South East Asia and then 3.1 events [HPD 0-5] from North West Europe (Fig. 1E). We found both importation and exportation events between the two European regions, with North West Europe having a mean of 8.3 [HPD 6-11] exportation events to South East Asia. To test the effect of including travel data for the Australian isolates, the same analysis was undertaken but instead, the sampling location was used, which suggested that the main importation event to Oceania was from North West Europe, rather than South East Asia (Supplementary Fig. 2B), an implausible migration route when travel history data were included. Collectively, these data provide strong evidence for multiple importation events of ST34 *Salmonella* 4,[5],12:i:- into Australia.

### Differential interruptions of the *fljAB* region are found in lineages of monophasic *Salmonella*

**Fig. 2:**
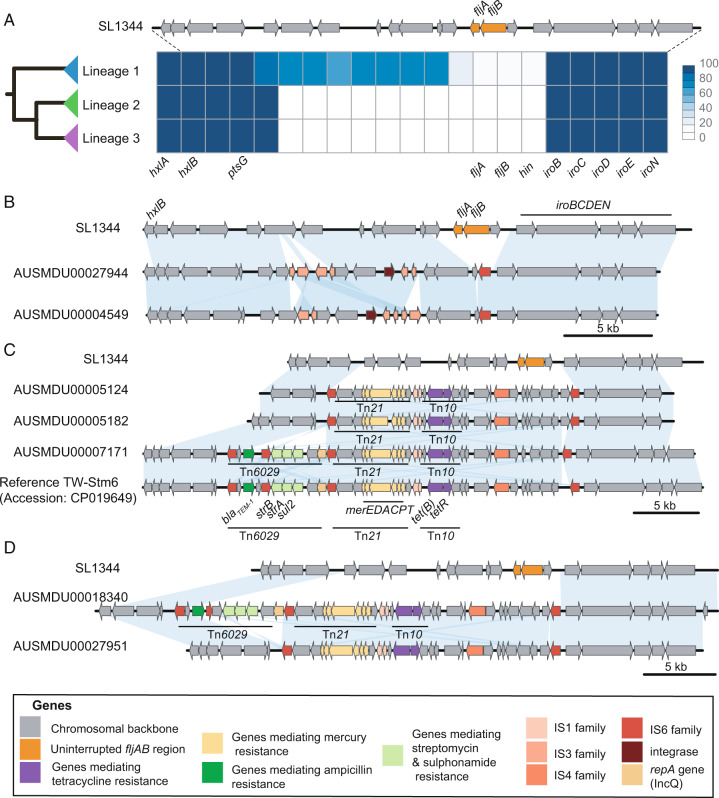
The interruption of the phase II flagella differs between lineages. **A** The frequencies that each gene in the phase II flagella region appears in each lineage is shown as a heatmap, with dark blue indicating the gene was detected in all isolates and white indicating that the gene was not detected in any. For comparison, genes in the biphasic ST19 *S*. Typhimurium SL1344 type are shown above. **B**–**D** The different interruption patterns of the phase II flagella in Australian *Salmonella* 4,[5],12:i:- genomes resolved through long-read sequencing are shown in comparison to the biphasic ST19 *S*. Typhimurium SL1344 type strain. The blue blocks indicate >95% sequence identity. **B** Two isolates from humans in Lineage 1. **C** Two isolates from humans and one bovine isolate from Lineage 2, in addition to the publicly available TW-STm6 isolate collected from a pig in 2014 and **D** Two isolates from humans in Lineage 3.

We explored the different patterns of gene interruption of the *fljAB* region in all isolates, observing that deletion and insertion events of mobile elements were more profound in Lineages 2 and 3 (Fig. 2 and Supplementary Fig. 3). The extent of *fljAB* interruptions, resulting in the monophasic profile, was characterized using three separate approaches (Supplementary Fig. 3).

In all three lineages, the interrupted flagellar region had a common insertion sequence (IS) element, IS*26* (part of the IS*6* family)^34^, located upstream of *iroB* with identical left direct repeat (DR_L_) sequences. The similarity of this insertion across all three lineages suggests this element represented an early integration event in the ancestral ST34 *Salmonella* 4,[5],12:i:- chromosome that has been maintained. Lineage 1 was characterized by deletion of *fljAB* genes and the two immediate flanking genes (putative transposase and DNA invertase *hin*), and the interruption of genes putatively classed as inner membrane proteins in the upstream region by IS from two families, IS*3* and IS*6* (Fig. 2A, B). Twelve Lineage 1 isolates were typed as *Salmonella* 4,[5],12:i:- from SISTR (an in silico serotyping approach)^35^, with ten of these phenotypically characterized as *Salmonella* 4,[5],12:i:-. The remaining three Lineage 1 isolates were typed in silico as *S*. Typhimurium, and were also characterized phenotypically as *S*. Typhimurium. This finding was supported by both alignment of short-read data to the *fljAB* regions and surrounding genes, and pangenome analysis for the presence/absence of these genes (Supplementary Fig. 3).

In contrast to Lineage 1, genomes from Lineage 2 and Lineage 3 had a more extensive chromosomal interruption with the deletion of surrounding genes encoding putative inner membrane, DNA/RNA helicase, and putative solute-binding proteins (Fig. 2A and Supplementary Fig. 3). The resolved chromosomal regions in members of these lineages were characterized by the region bounded by IS*6* family elements containing Tn*21* and a derivative of Tn*10* (Fig. 2C, D). The Tn*10-*like mobile element was associated with resistance determinants to tetracycline while the *merEDACPT* operon on Tn*21* was associated with resistance determinants to mercury (Fig. 2C, D). These observations suggest that the mobile elements replacing *fljAB* in Lineages 2 and 3 were acquired once and subsequently maintained within the population, with this event estimated to have occurred around ~2000 (HPD 1998–2002) (Supplementary Fig. 3A).

Further, the previously characterized Tn*6029* transposon^36,37^ was detected in some members of Lineages 2 and Lineages 3 (Fig. 2C, D). This element encodes resistance to ampicillin (*bla*_TEM-1_), sulfonamides (*sul2*), and streptomycin (*strAB*, also known as *aph(3”)-Ib* and *aph(6)-Id*), which together with the *tet(B)* gene carried on Tn*10*, confers the ASSuT profile associated with *Salmonella* 4,[5],12:i:-. The variable presence of Tn*6029* in the complete genomes from Lineages 2 and 3 demonstrates that this mobile element does not appear to be fixed in the chromosome. For example, Tn*6029* was found in two isolates from Lineage 2, from a human case and the publicly available isolate from a pig but was absent in the second human and cattle isolate. Lineages 2 and 3 were characterized by *tet(B)* mediating resistance to tetracycline carried on the chromosome with the remaining ASSuT genes integrated into the chromosome of some members of these lineages. This contrasted with Lineage 1 where the ASSuT profile was plasmid-mediated and all Lineage 1 isolates carried a *tet(A)* gene (Supplementary Fig. 4). Collectively, these data demonstrate stepwise interruptions of *fljAB*, with a successive accumulation of AMR genes that are distinct across the three major lineages.

### Acquired resistance determinants to third-generation cephalosporins and colistin further define ST34 *Salmonella* 4,[5],12:i:- lineages

**Fig. 3:**
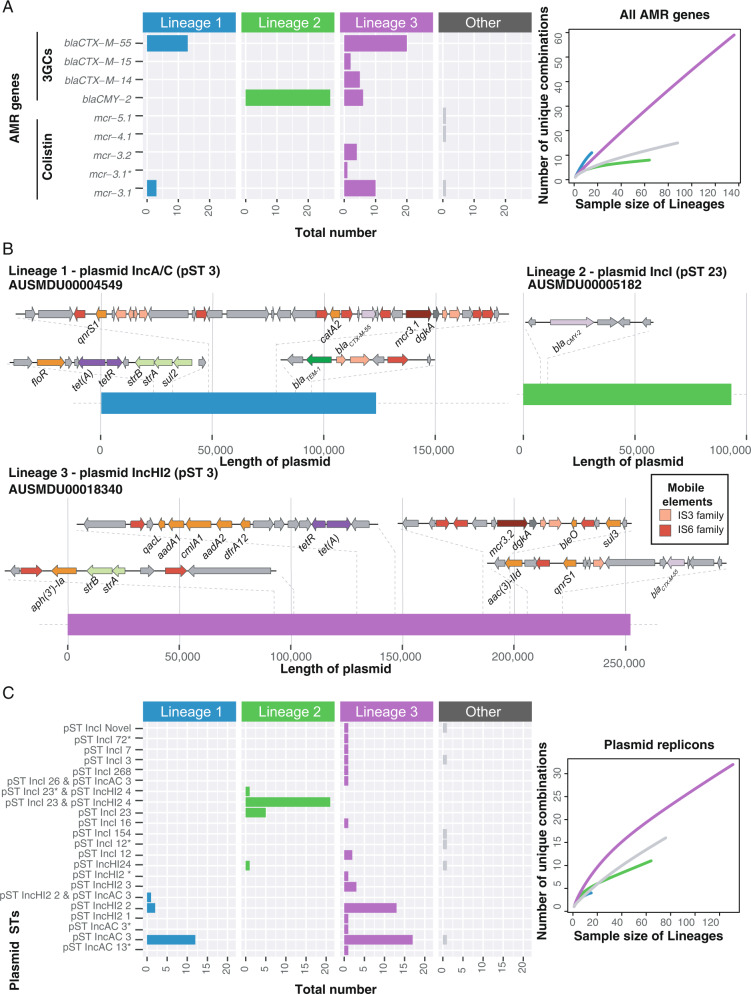
Third-generation cephalosporin and colistin resistance profiles differ by lineages and are associated with different plasmids. **A** AMR genes mediating resistance to third-generation cephalosporins (3GCs) and colistin was detected in the 309 isolates stratified by lineage membership. The * indicates a close but not exact sequence match. Rarefaction curves of the diversity in the unique AMR gene combinations for each lineage are shown to the right. **B** Regions encoding AMR in plasmids resolved from long-read data for common AMR profiles that mediate resistance to third-generation cephalosporins and /or colistin in the three lineages. **C** The plasmid STs in the genomes are shown by lineage membership for the top three plasmid replicons detected from the initial plasmid screen. The * indicates a close but not exact sequence match. Rarefaction curves of the diversity in the unique plasmid replicon combinations for each lineage are shown to the right. AMR antimicrobial resistance.

While the study inclusion criterion of exhibiting the epidemiological ASSuT profile for ST34 *Salmonella* 4,[5],12:i:- isolates resulted in nearly all isolates being considered MDR (defined as resistance determinants to three or more classes)^38^, extensive diversity was observed in the AMR profiles of all isolates (Supplementary Figs. 4 and 5). Genes mediating the ASSuT profile, *bla*_TEM-1_, *sul* genes, *strAB,* and *tet* genes, were found across the entire ST34 *Salmonella* 4,[5],12:i:- dataset (Supplementary Fig. 4), as were heavy metal resistance genes including those associated with mercury and tellurium resistance, and the *Salmonella* Genomic Island IV (SGI4), containing genes associated with resistance to silver, arsenic, and copper (Supplementary Figs. 4 and 6). However, there was also a diversity of mechanisms detected to clinically relevant antimicrobials including third-generation cephalosporins (3GC), colistin, and fluoroquinolones (Fig. 3 and Supplementary Fig. 4, Supplementary Data 1).

The *bla*_CMY-2_ gene was detected in 27/64 (42.2%) of the endemic Australian Lineage 2 isolates and was harbored on an IncI pST23 plasmid (Fig. 3, Supplementary Figs. 4 and 7). This IncI pST23 plasmid was highly similar (≥ 90% sequence identity) to a previously collected IncI plasmid from a *Salmonella* Newport strain (CVM 22462) isolated from a dog in 2003 in Arizona, USA^39^ (Supplementary Fig 7). Lineage 2 had relatively low AMR diversity compared to Lineages 1 and 3, reflected in both the number of drug classes to which the isolates were resistant and the number of unique AMR genes (Fig. 3A and Supplementary Fig. 4).

In both Lineages 1 and 3, which were associated with reported travel to South East Asia, the most frequently detected genes conferring resistance to 3GC and colistin were *bla*_CTX-M-55_ and *mcr-3.1* (Fig. 3). The *bla*_CTX-M-55_ gene was found in 13/15 (86.7%) of Lineage 1 isolates and 20/139 (14.3%) of Lineage 3 isolates, while *mcr-3.1* was found in 3/15 (20.0%) Lineage 1 isolates and 11/139 (7.9%) of Lineage 3 isolates (Fig. 3A). Lineage 3 had the greatest diversity of AMR profiles (Fig. 3A and Supplementary Fig. 4, Supplementary Data 1), and the greatest diversity of plasmid replicons (Fig. 3C). While the *bla*_CTX-M-55_ gene was only located on an IncAC pST3 plasmid in Lineage 1, in Lineage 3 it was also located on an IncH12 pST3 plasmid (Fig. 3B). The *mcr3.2* gene was detected in four Lineage 3 isolates, which co-occurred with IncHI2 pST3 in 3/4 isolates (Fig. 3E and Supplementary Fig. 2). In both IncAC and IncH12 plasmids (Fig. 3B), colistin resistance genes were part of a small transposable unit with *dgkA* (diacylglycerol kinase), flanked by IS elements previously shown to circularize and integrate into Enterobacterales genomes^40^. All colistin resistance genes in Lineage 3 co-occurred with *bla*_CTX-M-55_, likely on IncAC and IncH12 plasmids.

Of note, the plasmid-mediated *qnrs1* gene, which confers reduced susceptibility to ciprofloxacin, was detected in 10/15 (66.7%) of Lineage 1 and 32/139 (23.0%) of Lineage 3 isolates (Supplementary Fig. 4 and Supplementary Data 1). Point mutations in quinolone resistance determining regions (QRDRs) were only detected in three isolates (two from South East Europe and one from South East Asia) that were not members of any lineage, suggesting that QRDR point mutations are rare in ST34 *Salmonella* 4,[5],12:i:-.

### Lineage 2 and 3 ST34 *Salmonella* 4,[5],12:i:- isolates exhibit increased replication in phagocytic and non-phagocytic human cell lines compared with Lineage 1 isolates

To assess bacterial replication fitness, representative ST34 *Salmonella* 4,[5],12:i:- isolates Lineages 1–3, along with ST34 Lineage 1-matched biphasic *S*. Typhimurium isolates (Supplementary Table 1) were assessed for growth in nutrient broth and in a range of cultured human cells in vitro. Biphasic ST34 *S*. Typhimurium isolates express both flagellar antigens FliC and FljB, whereas the monophasic isolates only express FliC. Lineage 1 biphasic, as well as Lineage 2 and 3 monophasic isolates were comparable in their ability to replicate in nutrient broth over 24 h, with no statistical difference in growth between isolates within a lineage at all time points tested (Supplementary Fig. 8A, C, D). However, significant variation in growth rate was observed across Lineage 1 monophasic isolates (Supplementary Fig. 8B). Lineage 1 monophasic isolate, AUSMDU00004549 was significantly attenuated for growth in nutrient broth which may be attributed to the fact that it carries the most AMR genes of all six Lineage 1 isolates tested (Supplementary Fig. 8B and Supplementary Data 1). All Lineage 1 isolates contained the IncAC pST3 plasmid; however, the AMR profile varied across isolates (Supplementary Data 1).

**Fig. 4:**
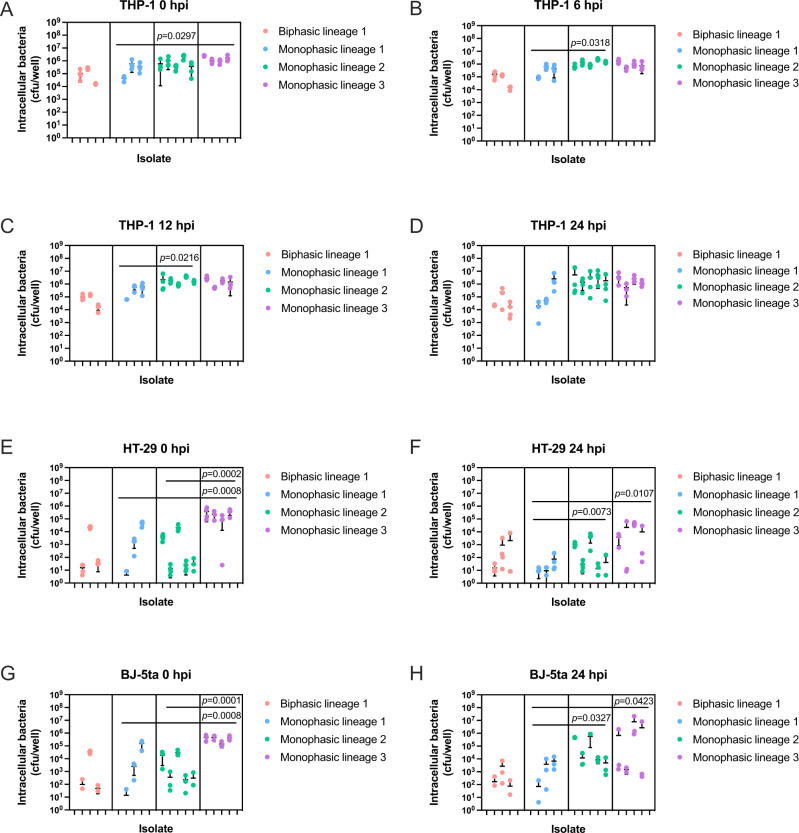
Lineage 2 and 3 ST34 *Salmonella* 4,[5],12:i:- demonstrate increased infectivity and replication in human cell lines. Differentiated THP-1 (human monocyte) cells (**A**–**D**), HT-29 (human intestinal epithelial) cells (**E**, **F**), or BJ-5ta (human fibroblast) cells (**G**, **H**) were infected at an MOI:10 with selected ST34 *Salmonella* 4,[5],12:i:- and ST34 matched-Lineage 1 biphasic *S*. Typhimurium isolates (Supplementary Table 1). THP-1 cells were lysed and intracellular bacteria enumerated as CFU/well at times 0, 6, 12, and 24 hpi. HT-29 and BJ-5ta cells were lysed and intracellular bacteria enumerated as CFU/well at times 0 and 24 hpi. Isolates depicted on the *x* axis correspond to isolates listed in Supplementary Table 1. Each dot represents CFU/well of a biological replicate (performed in technical duplicate), with error bars indicating ±1 standard deviation of *n* = 3 or 4 biological replicates. Statistical significance was determined by nested one-way ANOVA with Fisher’s uncorrected multiple comparisons test. MOI multiplicity of infection, hpi hours post-infection, CFU colony-forming units.

To investigate the in vitro intracellular replication dynamics of ST34 *Salmonella* 4,[5],12:i:- isolates, human macrophages (THP-1), human colonic epithelial cells (HT-29), and human fibroblasts (BJ-5ta) were infected with the same isolates tested for growth in nutrient broth (Supplementary Table 1). Both Lineage 1 monophasic and biphasic isolates were equally capable of infecting THP-1, HT-29, and BJ-5ta cells (Fig. 4A, E, G), and replication levels of these isolates were comparable across all cell types 24 h post-infection (Fig. 4B–D, F, H).

In contrast, Lineage 2 isolates replicated to significantly higher levels than Lineage 1 isolates in THP-1 cells over 12 h (Fig. 4B, C) and to significantly higher levels 24 h post-infection in HT-29 and BJ-5ta cells (Fig. 4F, H). Lineage 3 isolates infected all cell types more efficiently than Lineage 1 monophasic isolates (Fig. 4A, E, G) and infected both HT-29 and BJ-5ta cells more efficiently than Lineage 2 isolates (Fig. 4E, G). Lineage 3 isolates showed no significant replication advantage over lineages 1 and 2 in THP-1 cells (Fig. 4B–D) but exhibited significantly higher bacterial load in HT-29 and BJ-5ta cells compared to Lineage 1 monophasic isolates at 24 h post-infection (Fig. 4F, H). The growth of all individual bacterial isolates in each cell type is depicted in Supplementary Fig. 9A–L.

Of note, the higher bacterial load of Lineage 2 and 3 *Salmonella* 4,[5],12:i:- isolates did not negatively impact the viability of the cell types tested, with cellular cytotoxicity remaining comparable to biphasic Lineage 1 isolates over 24 h of infection (Supplementary Fig. 10A–E). Cytotoxicity was higher overall in THP-1 cells for all lineages, as expected in immune cells (Supplementary Fig. 10A–C), and significantly lower in HT-29 and BJ-5ta cells (Supplementary Fig. 10D, E). Collectively, these results demonstrate that Lineages 2 and 3 ST34 *Salmonella* 4,[5],12:i:- efficiently infect and survive in both phagocytic and non-phagocytic cells without compromising cellular viability.

## Discussion

Overall, our data demonstrate the co-circulation of distinct lineages of ST34 *Salmonella* 4,[5],12:i:- both in Australia and globally. Two lineages (Lineages 1 and 3) were associated with travel-acquired infections (predominantly from South-East Asia, a common travel destination from Australia), while one lineage (Lineage 2) was endemic to Australia. Our phylodynamic analyses are consistent with earlier reports of the global dissemination of the *Salmonella* 4,[5],12:i:- initially from Europe, then to Asia and the USA^11,12,41^. Importantly, our analyses explicitly included travel history data in our phylodynamic model, in the absence of which a different migration route would have been misleadingly supported. Our detailed genomic analysis of phase II flagellar interruption shows this key evolutionary event differentiates between lineages, with the integration and maintenance of Tn*21* and Tn*10* in Lineages 2 and 3 associated with a population expansion of these lineages over the past decade. IS elements were critical in mobilizing these transposons, and future research should investigate the influence of IS on the transcription of *fljAB* in monophasic and biphasic ST34 *Salmonella*. Our data also reveal distinct population dynamics relating to cephalosporin and colistin resistance within the three lineages. We hypothesize that differing AMR and plasmid profiles in ST34 *Salmonella* 4,[5],12:i:- reflect adaptation to distinct ecological niches, with potential differences in regional drug usage acting as a selective pressure for acquisition and retention of accessory genome content, as observed in other members of the Enterobacterales^42–44^.

Although the Australian endemic Lineage 2 isolates had the least AMR and plasmid diversity, ~40% of isolates in this lineage harbored an IncI pST23 plasmid carrying *bla*_CMY-*2*_, including all isolates from cattle. IncI plasmids have previously been associated with *Salmonella enterica* isolated from livestock species including cattle in the USA and poultry in Europe^11,45,46^. For example, cattle were identified as a key reservoir for *Salmonella* Newport strains harboring an IncI plasmid carrying *bla*_CMY-2_ in the USA in the early 2000s^45,46^, and two US isolates included in this study (one from cattle and one from food product)^11^ had *bla*_CMY-2_ co-occurring with IncI pST12. In this present study, isolates from Australian cattle and swine were closely associated with human isolates in Lineage 2, suggesting a possible livestock reservoir in Australia, with spill-over infections in humans. This hypothesis is supported by a previous molecular epidemiological study demonstrating endemic circulation of ST34 *Salmonella* 4,[5],12:i:- in Australian pig herds^47^. However, it is also plausible that the initial spill-over event was from humans to animals, with subsequent dissemination throughout both animal and human reservoirs. Of note, third-generation cephalosporin use in Australia is highly restricted, requiring a veterinary prescription^48^. Our finding of endemic circulation of a third-generation cephalosporin-resistant lineage amongst livestock and humans in Australia warrants further investigation, and further highlights the need for integrated, cross-sectoral AMR surveillance.

In contrast to Lineage 2, isolates in Lineages 1 and 3 had a diverse range of AMR profiles and plasmids and were associated with introductions from returned travelers. It is possible this diversity may reflect a range of host reservoirs in South-East Asia, where there are strong antimicrobial selection pressures for the emergence and spread of resistant Enterobacterales^42,43^. Large MDR IncAC pST3 and IncHI2 pST2 plasmids from *Salmonella* serovars (including *S*. Typhimurium and *Salmonella* 4,[5],12:i:-) have been previously reported from several parts of Asia^13,15,23^. In particular, resistance genes to 3GC (*bla*_CTX-M-55_) and colistin (*mcr3-1*) have been reported from human salmonellosis and livestock isolates from Asia^14,15,23,49^, and were identified in Lineages 1 and 3 in our study. Notably, US isolates included in this study formed a distinct subclade in Lineage 3, and were closely related to isolates collected from pigs in Vietnam^11,12^, further illustrating the propensity for global dissemination of AMR NTS.

In addition to the association of AMR profiles with host and geographic reservoirs, our data suggest that the successful emergence of *Salmonella* 4,[5],12:i:-, particularly Lineages 2 and 3, may also be driven by genetic factors that promote intracellular survival in host cells. Isolates from Lineages 2 and 3 were infected and replicated in both phagocytic and non-phagocytic human cells more efficiently than Lineage 1 monophasic and biphasic isolates, without inducing a significant increase in cellular cytotoxicity. Although AMR is clearly a major factor in the global spread of monophasic *Salmonella enterica*, in particular *S*. Typhimurium ST313, the leading cause of endemic NTS in Africa^50–52^, recent studies have also implicated loss-of-function mutations (genome degradation) in host-adaptation of *S*. Typhimurium ST313^10^. Further, a recent study showed that Lineage 2 isolates of *S*. Typhimurium ST313 escape early immune recognition by MAIT cells (mucosal immune cells), via overexpression of the bacterial enzyme RibB^53^. Similar to ST313, our initial observations suggest that *Salmonella* 4,[5],12:i:- Lineages 2 and 3 may have also evolved specific immune evasion mechanisms to maintain the viability of host cells and confer a competitive advantage over Lineage 1 isolates and possibly other biphasic NTS. These mechanisms could facilitate rapid and efficient bacterial dissemination of *Salmonella* 4,[5],12:i:- in human and animal hosts and may have potentially contributed to the population expansion of Lineages 2 and 3 and the subsequent decrease of Lineage 1 as reported here. A comprehensive future study of potential genome degradation events promoting bacterial virulence across ST34 *Salmonella* 4,[5],12:i:- lineages could provide valuable insights into not only functional relevance, but also the evolutionary trajectory and epidemiological relevance of loss- and gain-of-function mutations.

Although our data do not provide a direct causal link between the loss of phase II flagellin in *Salmonella* 4,[5],12:i:- and increased virulence potential, flagellin is a major pathogen-associated molecular pattern (PAMP) that potently activates host innate immune responses^54,55^. Recent studies on *S*. Typhimurium ST313 demonstrated a correlation between lower FliC expression, reduced inflammatory responses, and enhanced survival in macrophages; these phenotypes were linked to the high invasiveness and bacteremia associated with clinical *S*. Typhimurium ST313 infections^56–58^. One caveat of these studies, however, was that conclusions were drawn from the comparison of in vitro cellular responses between ST313 and ST19 isolates, two distinct lineages of NTS. Further, an association between flagellin deletion and increased invasiveness has been described in other host-adapted serovars, including *S. enterica* serovar Dublin in cattle and *S. enterica* serovar Gallinarium in poultry^59,60^. Overall, loss of flagellin expression appears to be a common theme in *Salmonella* serovars that cause invasive disease, a trait shared with ST34 *Salmonella* 4,[5],12:i:-. Although FljB may play a role in the ability of *Salmonella* 4,[5],12:i:- to evade host immune responses and survive inside host cells, further studies utilizing isogenic strains of biphasic ST34 *S*. Typhimurium deleted for *fljAB* are required to make causal associations of FljB loss with immune evasion and invasive capacity. Moreover, the extent of infectivity and replication differed both between and within ST34 *Salmonella* 4,[5],12:i:- lineages, suggesting that loss of FljB expression alone is not solely responsible for replicative fitness.

Our phylodynamic analyses represent one of the most diverse collections of ST34 *Salmonella* 4,[5],12:i:- to date, providing a snapshot of the global spread of this extensively drug-resistant pathogen. An important caveat of all phylogeographic analyses is that they are contingent on the sampling strategy across the actual geographic range of the organism in question. The availability of samples from currently unsampled locations will be important for our future understanding of the emergence of ST34 *Salmonella* 4,[5],12:i:-. Our data draws on Bayesian phylodynamics, microbial genomic epidemiology, and phenotypic analyses to investigate the origin, spread, adaptation, and replicative fitness of ST34 *Salmonella* 4,[5],12:i:- in Australia and provides a framework for ongoing surveillance of this important public health pathogen.

## Methods

### Setting and data sources

In Australia, the National Enteric Pathogens Surveillance Scheme (NEPSS) is a surveillance system for human, animal, and environmental enteric pathogens (including *Salmonella*) that has been operated by the Microbiological Diagnostic Unit Public Health Laboratory (MDU-PHL) at the University of Melbourne since 1978. Data in NEPSS include (i) epidemiological typing data, (ii) antimicrobial susceptibility data, (iii) phenotypic serovar and (iv) basic demographic data for human salmonellosis cases (e.g., age, gender, state of residence). When provided, travel history for patients with salmonellosis is also recorded in NEPSS.

### Sampling strategy

The final dataset comprised 309 genomes; these included 127 ST34 *Salmonella* 4,[5],12:i:- and nine *S*. Typhimurium (*n*=9) genomes from Australia, and 173 publicly available ST34 *Salmonella* 4,[5],12:i:- (Supplementary Data 1 and Supplementary Fig. 1A, B)

For Australian isolates, our sampling strategy involved two approaches. First, to ensure coverage of highly drug-resistant ST34 *Salmonella* 4,[5],12:i:-, we included all *Salmonella* 4,[5],12:i:- isolates that were phenotypically resistant to third-generation cephalosporins received at MDU-PHL over the study period. Second, to capture the diversity of ST34 *Salmonella* 4,[5],12:i:- over the study period, we included a random sample of Australian isolates from 2007-2017; these isolates also included ST34 *S*. Typhimurium that had the phenotypic AMR profile of ASSuT (a known epidemiological marker for ST34 *Salmonella* 4,[5],12:i:-). Six available ST34 *Salmonella* 4,[5],12:i:- isolates sourced from cattle were included. This gave a total of 136 Australian genomes included in this study; ST34 *Salmonella* 4,[5],12:i:- (*n* = 127) and ST34 *S*. Typhimurium (*n* = 9). All Australian isolates were sequenced at MDU-PHL (see below).

For publicly available isolates, our aim was to capture the diversity of ST34 *Salmonella* 4,[5],12:i:- circulating globally over the past decade by including isolates that formed part of several key studies reporting *Salmonella* 4,[5],12:i:-. To be included, isolates needed to have accessions for short-read data available, with geographic location (by country), year of collection, and source (human, food, or animal). To capture the diversity of *Salmonella* 4,[5],12:i:- circulating in the United States (US) from different sources, a random subset of isolates, stratified by human, animal, or food source, was included that was representative of lineages in the study from Elnekave et al.^11^. The final number of isolates comprised those from Italy (*n* = 13), the United Kingdom (*n* = 65), the US (*n* = 63), Australia (*n* = 2), and Vietnam (*n* = 30)^8,11,12,19^ (Supplementary Data 1).

### Whole-genome sequencing

Genomic DNA was extracted from a single colony using a QIAsymphony™ DSP DNA Mini Kit (Qiagen) according to the manufacturer’s instructions. Sequence libraries were prepared using NexteraXT with random library selection and whole-genome sequencing (WGS) was performed using Illumina NextSeq500, generating 150 bp paired-end reads.

Details of the included genomes are available in Supplementary Data 1. Genomes had a phred score 33, a depth of ≥50 relative to the reference genome, and filtering for isolates with the number of contigs ≤200 (filtering contigs <500 bases) (for details see assemblies below). The short reads of isolates sequenced at MDU-PHL are available on the NCBI Sequence Read Archive (BioProject PRJNA319593 or PRJNA556438).

### Phylogenetic analysis

The 309 genomes were mapped to the reference *Salmonella* 4,[5],12:i:- genome TW-Stm6 that was isolated from a pig fecal sample in Victoria, Australia in 2014^29^ (NCBI accession: CP019649) using Snippy (v4.3.7)/BWA MEM 1.2.0^61^ (https://github.com/tseemann/snippy). This reference was selected as it was a publicly available complete genome from Australia obtained during the study period. Single-nucleotide polymorphisms (SNPs) were called using Snippy (v4.3.7)/Freebayes (v1.0.2)^62^. Phage regions were identified using Phaster and masked from the alignment^63^. Recombinant regions were identified using Gubbins^64^ and removed from the 2713 base alignment. The final core alignment of 2693 bases was extracted with SNP-sites^65^. A maximum likelihood (ML) phylogenetic tree was inferred using IQ-TREE (v1.6.10) modeled using a general time-reversible (GTR) substitution model +Γ, including invariable sites as a constant pattern and 1000 ultra-fast bootstrap replicates^66^.

### Genome assemblies and screening

De novo assemblies of the genomes were constructed using Spades (v3.13)^67^. In silico multi-locus sequence types (STs) were determined using the program mlst with the *senterica* database (https://github.com/tseemann/mlst), and the in silico serovars of all genomes were determined using SISTR^35^. The genome assemblies of all isolates were screened for acquired AMR determinants using abriTAMR (https://github.com/MDU-PHL/abritamr) in conjunction with the NCBI AMRFinder database v3.2.1 (https://github.com/ncbi/amr)^68^. MDR isolates were those where mechanisms mediating resistance to three or more drug classes were detected. Known point mutations in quinolone resistance determining regions in *gyrA* and *parC* were investigated from the snippy output. Specifically, the variant calls for each isolate were investigated for non-synonymous point mutations in codons 83 and 87 in *gyrA* and codon 80 in *parC*. Known plasmid replicons were screened using ABRicate (https://github.com/tseemann/abricate) against the PlasmidFinder database^69^, with a minimum identity and minimum coverage thresholds of 95%. The diversity of unique combinations of AMR genes and plasmid replicons was assessed using the R package vegan (v2.5-6)^70^. The input was the number of unique combinations of either AMR genes or plasmid replicons detected in each of the three lineages or other for isolates that were not a member of one of the three lineages. These data were visualized with the rarecurve function. The number of AMR genes belonging to different drug classes (Supplementary Data 1) was plotted using ggplot2 (v3.2.1)^71^ by both lineage membership and geographical membership.

### Long-read sequencing and complete genome assembly

Seven Australian genomes underwent long-read sequencing, three with PacBio and four with Oxford Nanopore (see Supplementary Data 2). Isolates were selected for sequencing based on the diversity of AMR profiles and lineage membership. PacBio data was sequenced and assembled as previously described^72^. Briefly, raw sequence data were assembled using HGAP v3 (SMRT- Portal v2.3.0)^73^ with default parameters except for seed length set to 1 kb and genome size to 5 Mb. For isolates sequenced with the Oxford Nanopore GridION X5, genomic DNA (gDNA) of isolates was prepared from solid media scrapings of pure culture using the Genelute Bacterial Genomic DNA Kit (Sigma-Aldritch), and the Gram-negative protocol. Libraries were prepared through a 1D native barcoding genomic DNA approach (SQK-LSK109), and sequenced on an R9.4 flowcell before basecalled using Guppy (3.1.5) and samples de-multiplexed using Porechop (v0.2.4) (https://github.com/rrwick/Porechop), using the --require_two_barcodes option. The long reads were first filtered for minimum length 1 kb and target bases 5 Mb using Filtlong (v0.2.0) (https://github.com/rrwick/Filtlong), then assembled using both the hybrid mode of Unicycler^74^ and Flye^75^. Post-assembly comparison, the Unicycler outputs were kept as this approach produced complete genomes for all four isolates, with the chromosome structure, and plasmid type and size, consistent for each respective lineage. Genomes were annotated with Prokka (v1.14.0)^76^. The complete assemblies of the PacBio isolates and Oxford Nanopore long-read data are available at the European Nucleotide Archive (ENA) under PRJEB41036.

### Phylogeographical reconstruction of Salmonella 4,[5],12:i:-

To investigate temporal signal in the ST34 *Salmonella* 4,[5],12:i:- genomes, we first used TempEst (v1.5)^77^. A regression analysis was performed of the root-to-tip branch distances within the ST34 ML phylogeny as a function of year of collection, using the heuristic residual mean squared method and to select the best-fitting root. The resulting data was visualized in R using ggplot2^71^.

The final alignment was analyzed in BEAST v1.10.4^78^. We calibrated the molecular clock using including isolation dates for each genome (by year of collection) and specified locations based on major geographical regions (defined by the Australian Bureau of Statistics 1269.0 Standard, 2016). A GTR+Γ substitution model was specified with different combinations of molecular clock models (strict or uncorrelated relaxed with an underlying lognormal distribution) and tree priors (constant coalescent or exponential growth coalescent). Each model was fit using a Markov chain Monte Carlo of 200 million iterations, sampling every 20,000 iterations. We assessed sufficient sampling from the stationary distribution by verifying that the effective sample size (ESS) of key parameters was at least 200. We assessed convergence by repeating the analyses and verifying that they reached the same stationary distribution. Because our alignments consisted of SNPs only, we specified the number of constant nucleotides in the model.

We assessed statistical support for models by approximating their log marginal likelihood using generalized stepping-stone (GSS) and calculating the Bayes factors^79^. The ESS of key parameters was>200. The highest supported model was the relaxed lognormal clock with a coalescent exponential population model. We used the Bayesian evaluation of temporal signal (BETS) method to explicitly assess temporal signal under the Bayesian framework^30^ in Beast v1.10.4^78^ with the best-fitting model parameters with and without sampling dates. The premise of this approach is to test two competing models, one with and one without sampling times using their log marginal likelihood.

The frequency of importation or exportation events into Australasia was inferred using a discrete phylogeographic approach (i.e., a migration model), also known as Bayesian stochastic mapping, as implemented in Beast 1.10.4^78^. Our locations consisted of the five major geographical regions (defined by the Australian Bureau of Statistics 1269.0 Standard, 2016) from which the isolate was collected. These five geographical regions were the Americas, North West Europe, Oceania, South East Asia, and South East Europe. To exploit available metadata, we included travel history data from returning Australian travelers under the highest supported model selected above. This approach consists of specifying singleton internal nodes with the travel origin as their location for the Australian isolates (where there was reported travel). For comparison, we also conducted these analyses without such travel history data (using Oceania as the sampling location)^33^. We assessed convergence of the MCMC and sufficient sampling as above. We inferred the posterior number of migration events between the five possible regions and the amount of time spent at each state, known as Markov rewards. These data were visualized in R using circlize v0.4.8^80^.

We obtained the maximum-clade credibility tree (MCC) using TreeAnnotator v1.10.4 using the keep heights option from the run with the highest ESS values where the reported travel history for the Australian isolates was included. The final MCC tree was visualized in R using ggtree v1.16.6^81^. To investigate the lineages in circulation in Australia, three lineages were defined using the getMRCA function in ape v5.3^82^ for each of the three groups with >10 Australian isolates.

### Modeling effective population size within Australian lineages

The effective population size (*Ne*) of the three *Salmonella* 4,[5],12:i:- lineages was modeled using SkyGrowth^83^. To address the population dynamics of the three lineages in Australia, phylogenies were extracted from the MCC phylogeny using ape^82^ for each of the three lineages circulating in Australia. These three trees were used as input for Skygrowth^83^, with *res* parameter equal to an Ne changing every month for nine years in Lineage 1 and eleven years in Lineages 2 and 3 and a *tau0* smoothing parameter of 0.1.

### Interruption of the fljAB region

To explore the interruption of the *fljAB* region in both public and Australian data, chromosomal regions surrounding the phase II flagellar region were extracted from the publicly available genomes *S*. Typhimurium SL1344 (accession: NC_016810) and the complete Australian genomes using seqret in EMBOSS (v6.6.0)^84^. BLAST comparisons were undertaken and visualized in R using GenoPlotR (v0.8.9)^85^ to show regions with ≥ 95% sequence homology.

The coverage of phase II flagellar region in all isolates was explored in detail using two complementary approaches. First, all isolates were mapped to the publicly available ST19 SL1344 (accession: NC_016810) type strain using Snippy (v4.6.0) with all regions masked except the phase II flagellar region (shown in Fig. 2) and the percentage coverage of reads across the genomic region determined. Second, the presence/absence of genes in the phase II flagellar region in all isolates was explored using Panaroo (v1.2.4)^86^. The gff files of the annotated assemblies were used as input for Panaroo to cluster the pangenome using strict clean-mode and default parameters. The protein sequences for the annotated genes from the SL1344 phase II flagellar region were used to identify the gene clusters from panaroo in the Australian isolates typed as Typhimurium from SISTR. The presence/absence of these genes in all isolates was extracted from the Panaroo output tables. The individual genes for each isolate were plotted against the MCC tree using ggtree^81^. The frequencies of the individual genes within each of the three lineages were visualized in R using pheatmap v1.0.12 (https://cran.r-project.org/web/packages/pheatmap/)

### Characterization of plasmids

Plasmids were further characterized by plasmid multi-locus sequence typing (pMLST) for three plasmid replicons detected most frequently within the isolates, specifically IncA/C, IncHI2, IncI. The schemes for these plasmid types were obtained from https://pubmlst.org/plasmid/ and formatted for use with ARIBA (v2.14.1)^87^. The IncI replicon plasmid from AUSMDU00005182 was then compared to publicly available data using BLAST and found to be homologous to *Salmonella* Newport pCVM22462 (Accession CP009566). The homology was visualized using GenoPlotR^85^. Further investigation of the IncI plasmid was undertaking by mapping all isolates to the complete AUSMDU00005182 genome from Lineage 2 and the presence of the same IncI plasmid was inferred if reads mapped to ≥ 95% of the reference. The regions encoding AMR genes on the plasmids were resolved by long-read sequencing and plotted in R with GenoPlotR^85^.

### Screening for elements associated with heavy metal resistance

Genome assemblies were screened for the SGI4^8^ (SO4698-09, accession LN999997) with blastn. SGI4 was determined as being present with >90% coverage and >99% identity to the reference. Genes mediating heavy metal resistance were identified from the AMRFinderPlus database v3.2.1 and screened with ABRicate using a minimum identity of 75% and minimum coverage of 90%. The resulting data were transformed in binary presence/absence data in R using tidyverse (v1.2.1)^88^.

### Selection of isolates for use in phenotypic assays

Between 3-5 representative *Salmonella* 4,[5],12:i:- isolates were selected for phenotypic characterization from each Lineage 1, 2, and 3, along with three Lineage 1-matched biphasic isolates. All isolates selected for phenotypic analysis were utilized for complete genome assembly and are represented in the phylogenetic trees depicted in Fig. 1 and Supplementary Figs. 3, 4. Further, at least two of each monophasic Lineage 1, 2, and 3 isolates were used in additional genomic analyses represented in Figs. 2B, C and 3B. The isolates selected are listed in Supplementary Table 1 and details of plasmid and AMR profiles for each isolate are listed in Supplementary Data 1.

### Phenotypic comparison of growth rates in liquid culture medium

Isolates were plated onto Luria Bertani (LB) agar and incubated at 37 °C overnight (+50 μg/ml w/v streptomycin for SL1344). For CFU/ml readings in broth culture, single colonies of each bacterial isolate were inoculated into 10 ml of LB broth and incubated in a shaking 37 °C incubator at 200 rpm for ~16 h. From these cultures, 1/100 starter cultures (5 ml total) were inoculated and incubated for 3–4 h as above. Following the 3–4 h incubation, all cultures were standardized to OD_600_ 0.05 and 200 µl of each culture was added to a 96 well plate in duplicate and grown for 24 h in a BMG FLUOstar Omega heated to 37 °C and shaking at 200 rpm. The plate reader was stopped for no longer than 3 min to remove 10 µl of culture from each well using a multichannel pipette at times 0, 3, 6, 9, 12, and 24 hr. Serial dilution of the culture was performed in PBS and plated onto LB agar in duplicate. This experiment was performed three independent times (biological triplicate) on separate days. Differences between isolates at each time point were determined by two-way ANOVA or mixed-effects analysis with Tukey’s multiple comparisons post-test (GraphPad Software v9.0). Statistical significance was determined to be *p* < 0.05.

### Phenotypic comparison of growth rates in and human cell lines

Human macrophage (THP-1) (ATCC^®^ TIB-202™) and colonic epithelial (HT-29) (ATCC^®^ HTB-38™) cell lines were maintained in Roswell Park Memorial Institute (RPMI) 1640 media + 200 mM GlutaMAX (Life Technologies) supplemented with 10% v/v Fetal Bovine Serum (Bovogen) and grown in a humidified 5% CO_2_ 37 °C incubator. Human h-TERT immortalized foreskin (BJ-5ta) (ATCC^®^ CRL-4001™) fibroblasts were maintained in Dulbecco’s Modified Eagle Medium (DMEM) containing l-glutamine (Life Technologies) and 10% FCS, 50 ng/ml hygromycin. Prior to infections, THP-1 cells were differentiated for 3 days with 25 ng/ml phorbol 12-myristiate-12 acetate (PMA, Sigma-Aldrich). For intracellular replication experiments, single colonies of each bacterial isolate (Supplementary Table 1) were inoculated separately (triplicate) in LB broth and incubated overnight at 37 °C at 200 rpm, then sub-cultured (at 1/100) for 3 h in the same conditions in 5 ml LB. Mammalian cells were infected at a multiplicity of infection (MOI) of 10 in RPMI or DMEM (depending on cell type) in duplicate and centrifuged at 525×*g* to synchronize bacterial uptake. Following 30 min incubation at 37 °C in 5% CO_2_, cells were incubated with 100 μg/ml gentamicin in RPMI for 1 h to inhibit extracellular bacterial growth in the media, then replaced with 10 μg/ml gentamicin in 1% v/v FBS/RPMI or FBS/DMEM for the remainder of the infection. For THP-1 cells, samples were collected at times 0-, 6-, 12- and 24-h post-infection. For HT-29 and BJ-5ta cells, samples were collected at 0- and 24-h post-infection. All Lineage 1 and 2 isolates were sensitive to gentamicin. We observed that all lineage 3 isolates were resistant to gentamicin which resulted in overgrowth of these isolates in the tissue culture media. Therefore, for all Lineage 3 isolates, tissue culture media was supplemented with 0.5 µg/ml meropenem (to which Lineage 3 isolates were susceptible) from 6 h post-infection (in addition to the gentamicin treatment applied to Lineages 1 and 2 isolates) to restrict extracellular bacterial growth. Previous work suggests meropenem has limited ability to cross the plasma membrane at very low concentrations (0.5 µg/ml)^89^. To confirm this, we assessed the intracellular viability of isolates with either gentamicin alone or gentamicin in combination with meropenem and found no difference (Supplementary Fig. 11).

Cell viability was measured by lactate dehydrogenase (LDH) release into the supernatant of infected cells. Here, cell supernatant was collected before cell lysis and LDH release was quantified as per the manufacturer’s instructions (Promega). The percentage of cytotoxicity was calculated by comparison to LDH release from 100% lysed uninfected control cells. For enumeration of intracellular bacteria, media was removed, and cells were washed twice with PBS to remove extracellular bacteria, followed by lysis in 0.1% Triton X-100. Lysates were serially diluted in PBS in duplicate and plated onto LB agar. Bacterial enumeration and cell viability assays were performed over 3–4 biological replicates, each performed independently, with a new passage of host cells, on a separate day. Within each biological replicate, technical duplicates were performed for each isolate. All statistical analyses were performed using Prism software (GraphPad Software v9.0) and determined by two-way ANOVA with Tukey’s post-test for multiple comparisons. Statistical significance was determined to be *p* < 0.05. To compare groups of isolates between lineages, we performed a nested one-way ANOVA with Tukey’s multiple comparisons test, which allowed for the inclusion of individual isolates in sub-groups of one lineage, and then the comparison between the different lineages.

### Reporting summary

Further information on research design is available in the Nature Research Reporting Summary linked to this article.

## Supplementary information

Supplementary Information

Reporting Summary

Description of Additional Supplementary Files

Supplementary Data 1

Supplementary Data 2

## Data Availability

Supplementary Data 1 lists the individual accessions for all isolates, with associated metadata. Short-read data for Australian isolates in this study are available from the NCBI Sequence Read Archive (BioProject PRJNA319593 or PRJNA556438). Long-read data are available at the European Nucleotide Archive (ENA) under PRJEB41036, and individual accessions are provided in Supplementary Data 2. An interactive annotated phylogeny is available in Microreact https://microreact.org/project/mfxxBchBsUpsJu7nvfkFw4. Antimicrobial resistance genes were detected using AbritAMR (https://github.com/MDU-PHL/abritamr) in conjunction with the AMRFinder database v3.2.1 (https://github.com/ncbi/amr). Source data for phenotypic work are provided with this paper. Data supporting the findings of this study are available within the text and in Supplementary files. Source data are provided with this paper.

## Source data

Source Data
