# Supplementary material for: Evolutionary dynamics of multidrug resistant Salmonella enterica serovar 4,[5],12:i:- in Australia
Source: Nat Commun. 2021 Aug 9;12:4786. doi: 10.1038/s41467-021-25073-w (PMC8352879; doi:10.1038/s41467-021-25073-w)
Supplement: Supplementary file 1 — Supplementary Information [file 41467_2021_25073_MOESM1_ESM.pdf]

Evolutionary dynamics of multidrug resistant *Salmonella enterica* serovar 4,[5],12:i:- in Australia

Danielle J. Ingle, Rebecca L. Ambrose, Sarah L. Baines, Sebastian Duchene,
Anders Gonçalves da Silva, Darren Y. J. Lee, Miriam Jones, Mary Valcanis,
George Taiaroa, Susan A. Ballard, Martyn D. Kirk
Benjamin P. Howden, Jaclyn S. Pearson, Deborah A. Williamson

Supplementary Note

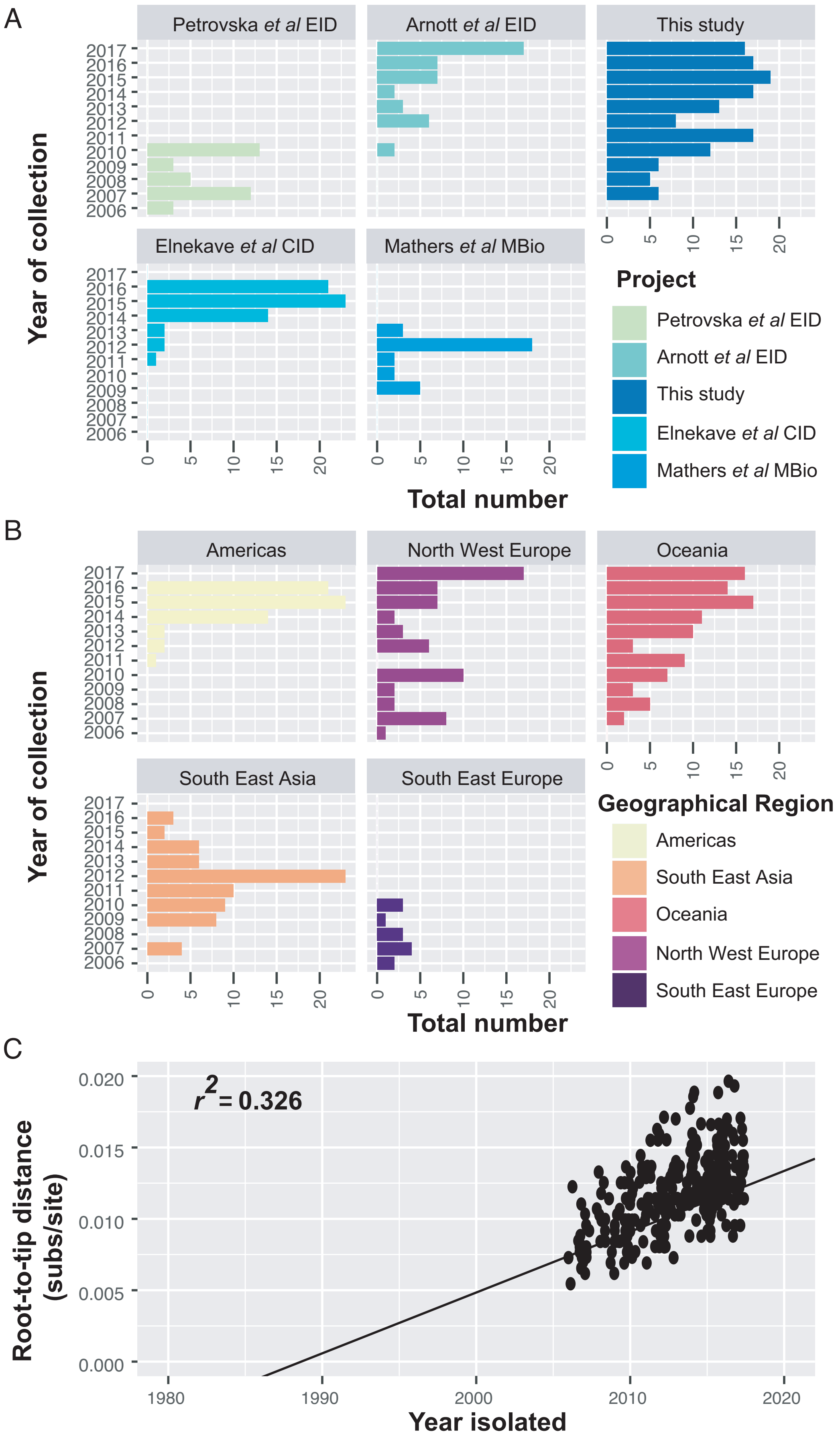

A
Petrovska et al EID
Arnott et al EID
This study
Elnekave et al CID
Mathers et al MBio
Year of collection
Total number
Project
Petrovska et al EID
Arnott et al EID
This study
Elnekave et al CID
Mathers et al MBio
B
Americas
North West Europe
Oceania
South East Asia
South East Europe
Year of collection
Total number
Geographical Region
Americas
South East Asia
Oceania
North West Europe
South East Europe
C
$r^2 = 0.326$
Root-to-tip distance (subs/site)
Year isolated

Supplementary Figure 1: Distribution of sampling times and root-to-tip regression

A) Summary of the source of the 309 genomes included in this study. The reported year of collection is on the x axis. **B)** Geographic sampling coverage of the 309 genomes across five geographical regions. The reported year of collection is on the x axis. **C)** Tempest regression of root-to-tip distance as a function of sampling time. The slope is a crude estimate of the substitution rate for the recombination-free SNP alignment, with the x-intercept indicative of the age of the root node and the r^2 is a measure of clocklike behavior.

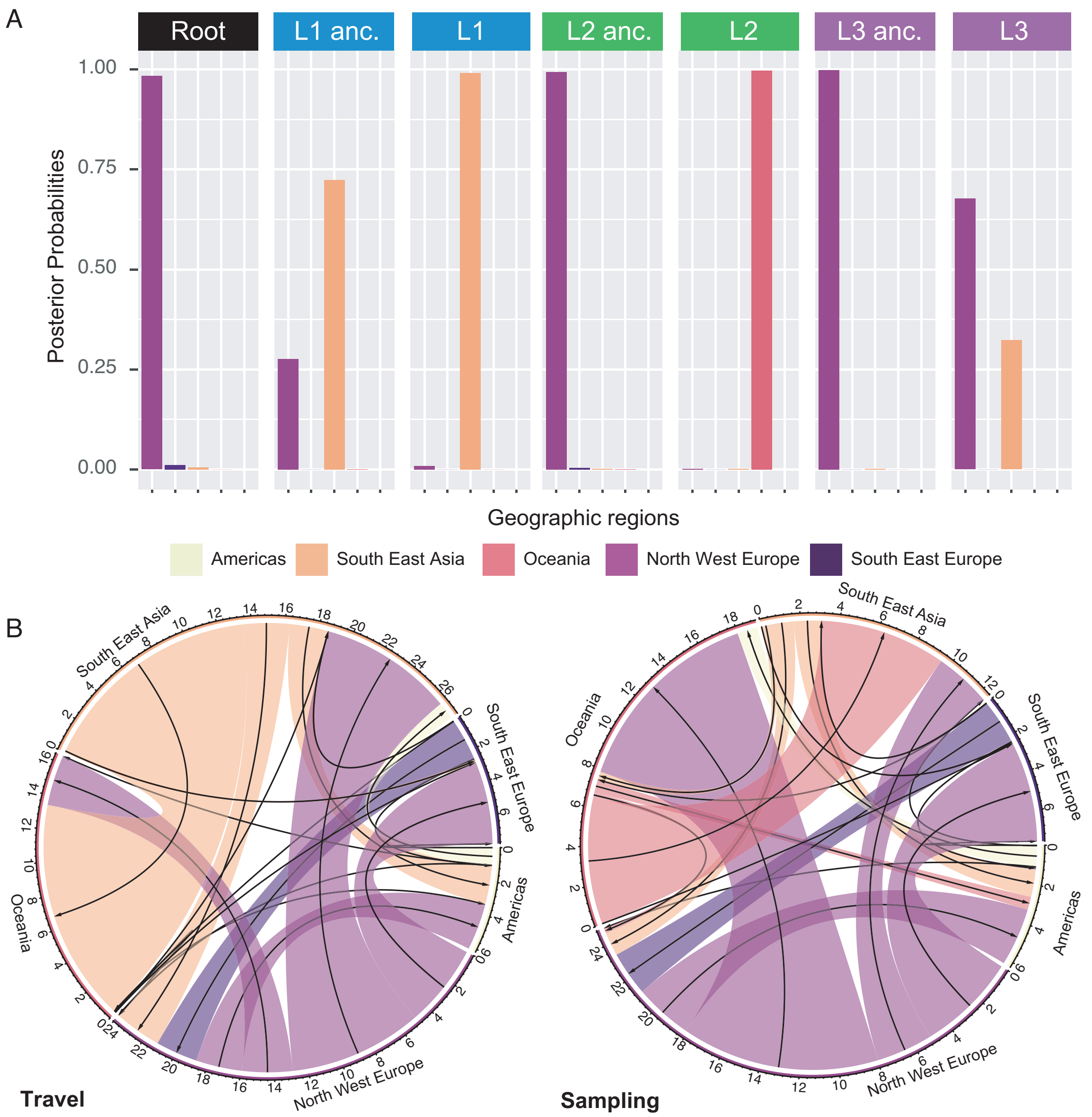

Supplementary Figure 2: Phylogeography and migration rates for sampling and travel

A) The posterior probability of geographical locations for individual nodes in the MCC shown in Figure 1A. These include the root, MRCA for each lineage and the ancestral locations of the three Australian lineages are shown. **B)** Circular migration diagrams of migration events between the five geographical regions using both reported travel in the Australian data (to South East Asia, duplicated from Figure 1E) and sampling location. The size of the colored block denotes the posterior mean number of inferred migration events from the Bayesian phylogeographical analysis and arrows denote directionality. MCC; maximum-clade-credibility, MRCA; most recent common ancestor

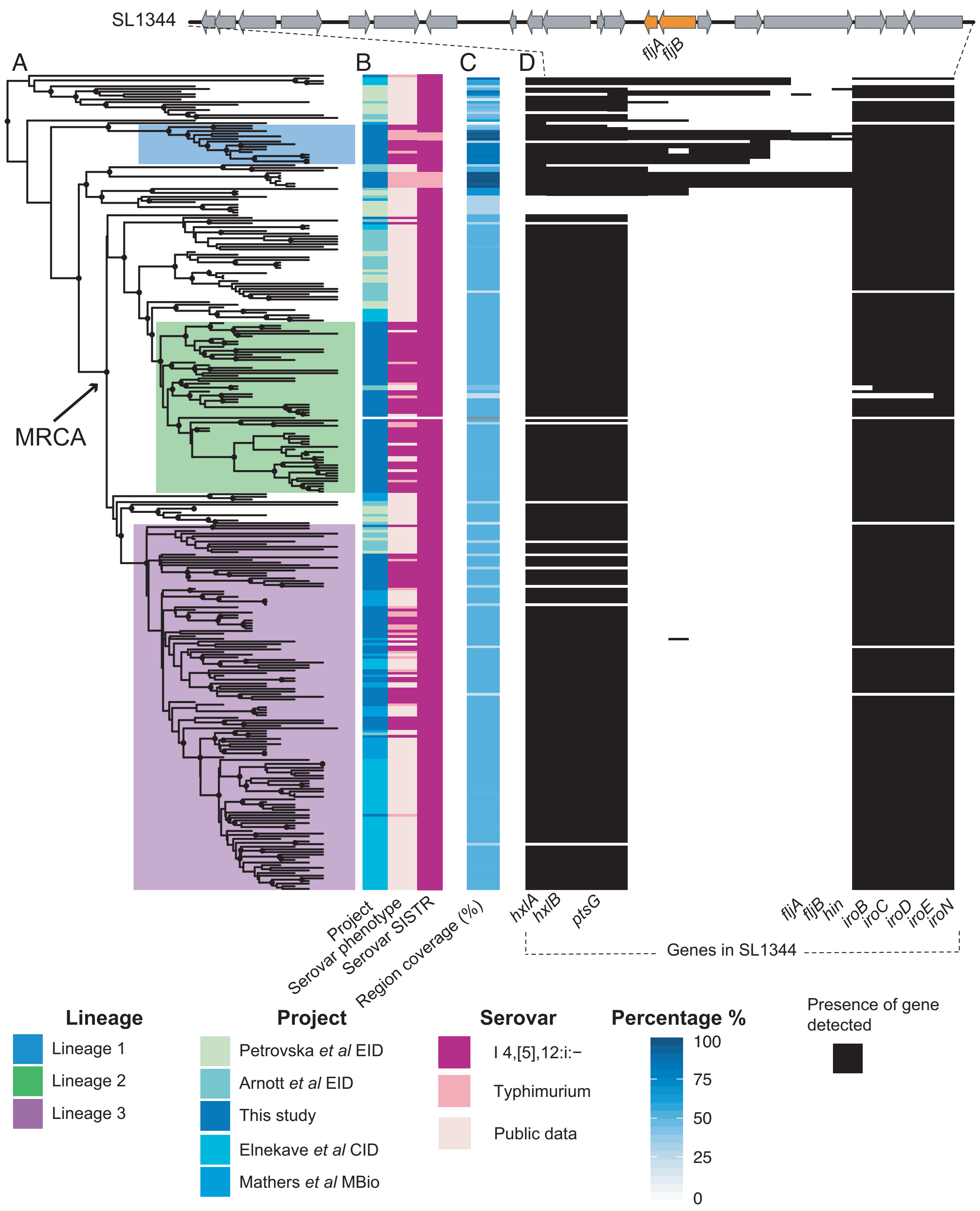

Supplementary Figure 3: Summary of different patterns of interruption of phase II flagella in relation to the *Salmonella* 4,[5],12:i:- phylogeny

A) The inferred maximum clade credibility tree with membership of the three defined ST34 *Salmonella* 4,[5],12:i:- lineages. The MRCA for lineages 2 and 3 is shown. **B)** The study from which the isolates were sourced, the reported phenotypic serovar (for the Australian data only; the international isolates are 'public data') and the inferred serovar from SISTR are

all shown to the right of the tree. **C)** The percentage of the phase II flagella region and surrounding housekeeping genes that were covered by reads aligning to the region is shown as a heatmap for each isolate. **D)** the presence /absence of genes in the SL1344 reference genome based on the pangenome analysis using Panaroo. MRCA; most recent common ancestor

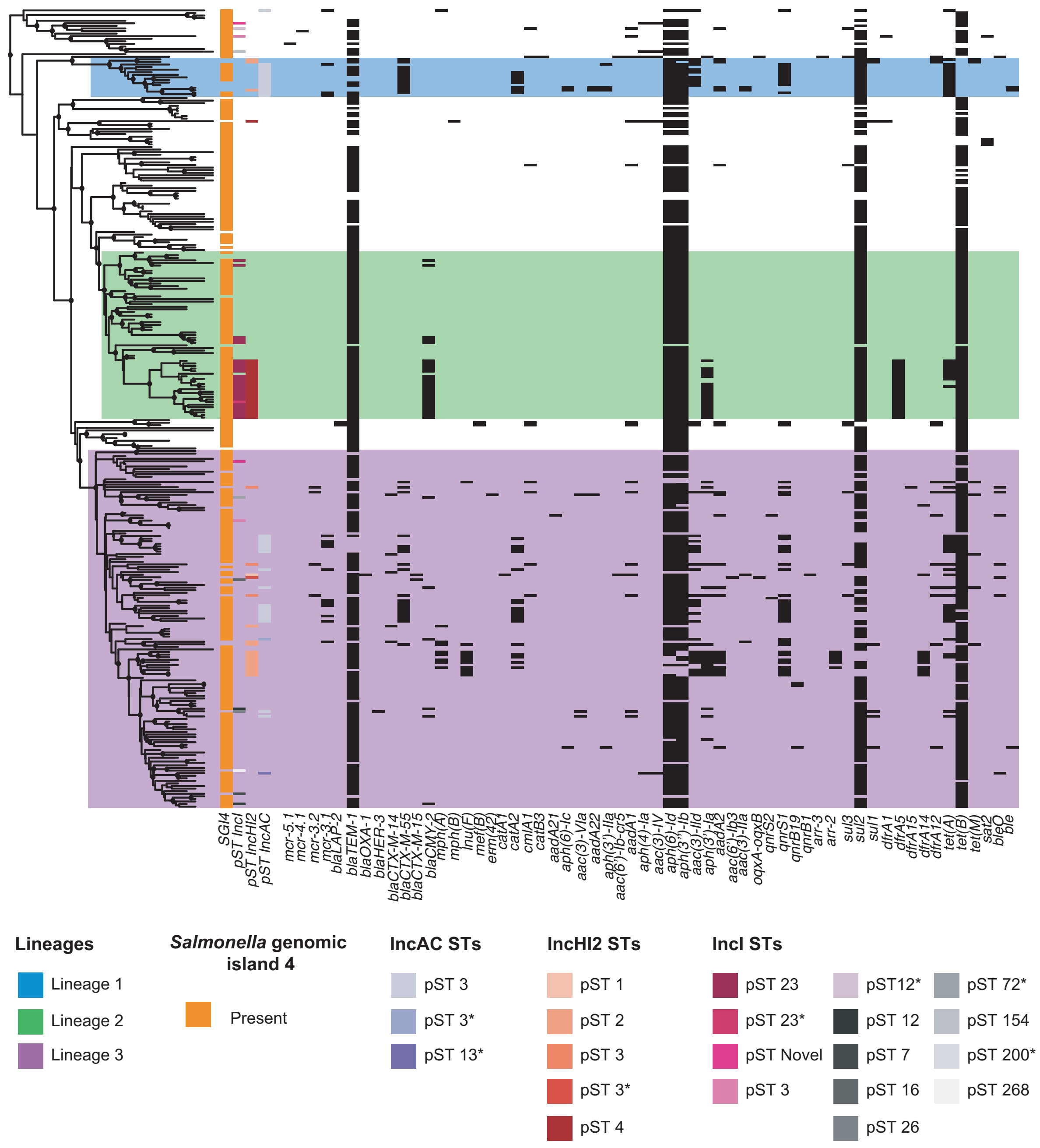

Supplementary Figure 4: Profiles of accessory genomic content mediating resistance to antimicrobials, SGI4 and plasmids.

The inferred maximum clade credibility phylogeny for the 309 genomes is shown to the left with three lineages highlighted as colored boxes extending through the figure. The presence of the SGI4, presence of pST for the top three plasmid replicons detected, IncI, IncAC and IncHI2 plasmids, and a binary heatmap for the presence/absence of genes mediating antimicrobial resistance with black indicating the gene was detected in an isolate. The legend is shown to the right of the figure. SGI4; *Salmonella* Genomic Island IV, pST; plasmid sequence type

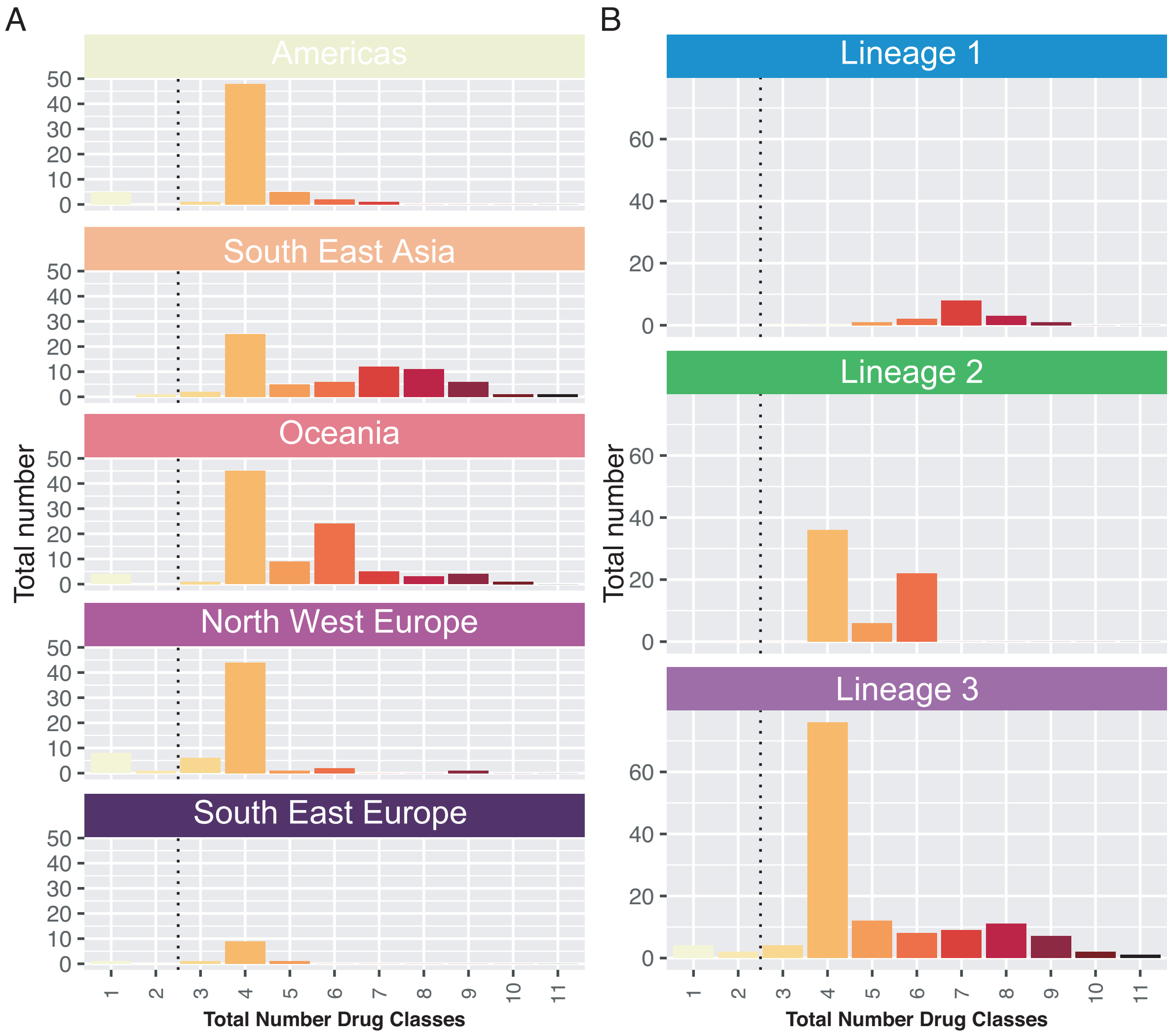

Supplementary Figure 5: Summary of multidrug resistance profiles stratified by geographic region and lineage. **A)** The number of known AMR genes detected that mediate AMR to different drug classes by geographical region (using inferred location of infection from the Australian isolates from cases with reported international travel) and **B)** the number of AMR genes detected in isolates belonging to the three ST34 *Salmonella* 4,[5],12:i:- lineages (Lineage 1 n = 15 , Lineage 2 n = 64, Lineage 3 n = 139). AMR; antimicrobial resistance

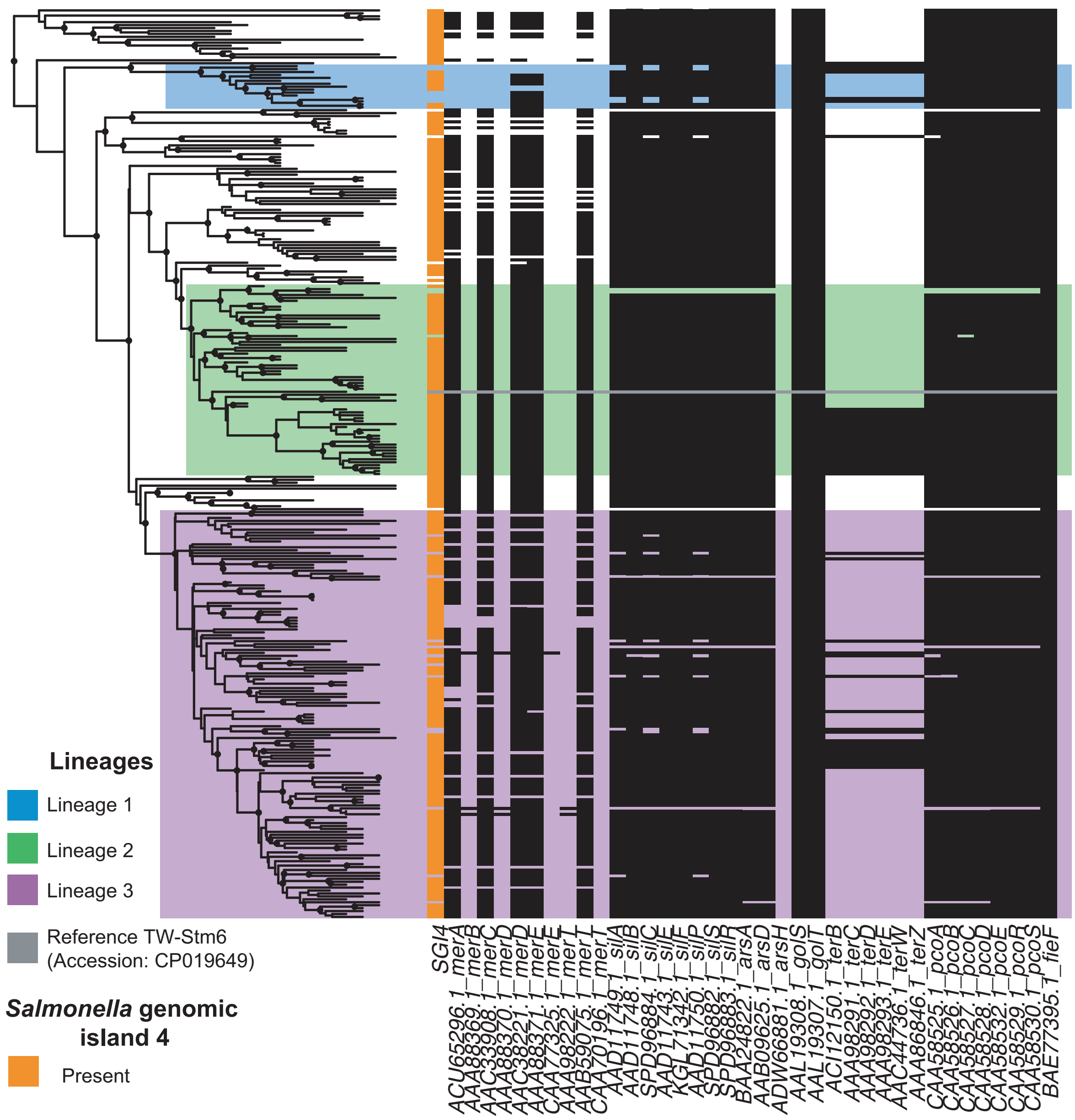

Supplementary Figure 6: Profiles of accessory genomic content mediating resistance to heavy metals.

The inferred maximum clade credibility phylogeny depicting the three key in the colored boxes extending through the figure. The presence of SGI4 is shown to the right of the phylogeny in orange. The presence/absence of genes associated with heavy metal resistance are shown as a heatmap to the right of the phylogeny. SGI4; *Salmonella* Genomic Island IV.

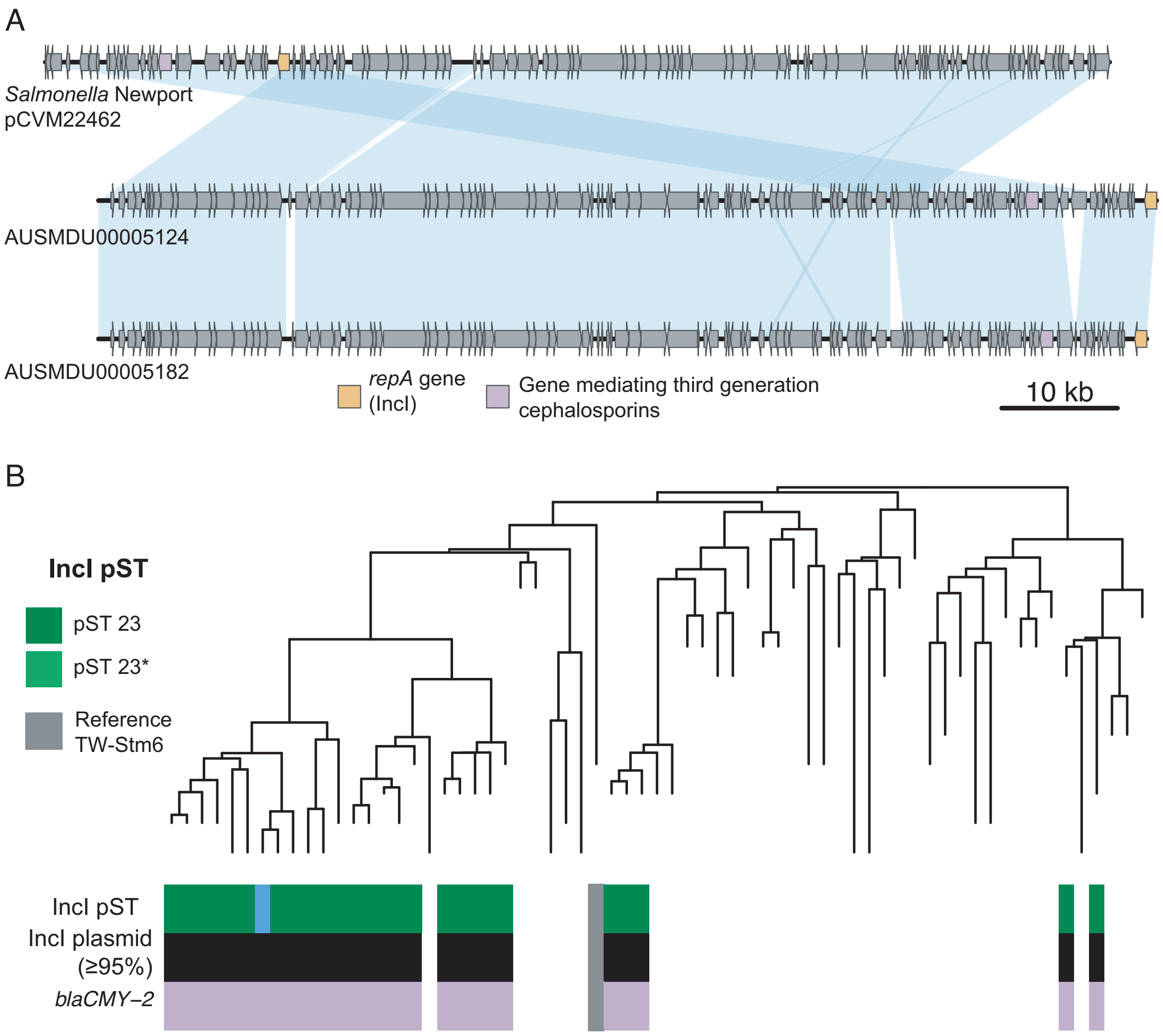

Supplementary Figure 7: Comparison of IncI plasmid from *Salmonella* Newport detected in Australian Lineage 2 isolates.

A) Visualization of sequence homology between three IncI plasmids, one from *Salmonella* Newport and two Australian *Salmonella* 4,[5],12:i:-. The blue shading between the two plasmids indicates ≥90% of nucleotide homology between the isolates. **B)** Dissemination of IncI plasmid (as determined by ≥95% of coverage of plasmid reference) within Lineage 2. The pST for IncI and the presence/absence of *bla*$_{\text{CMY-2}}$ mediating resistance to third generation cephalosporins are also shown. pST; plasmid sequence type

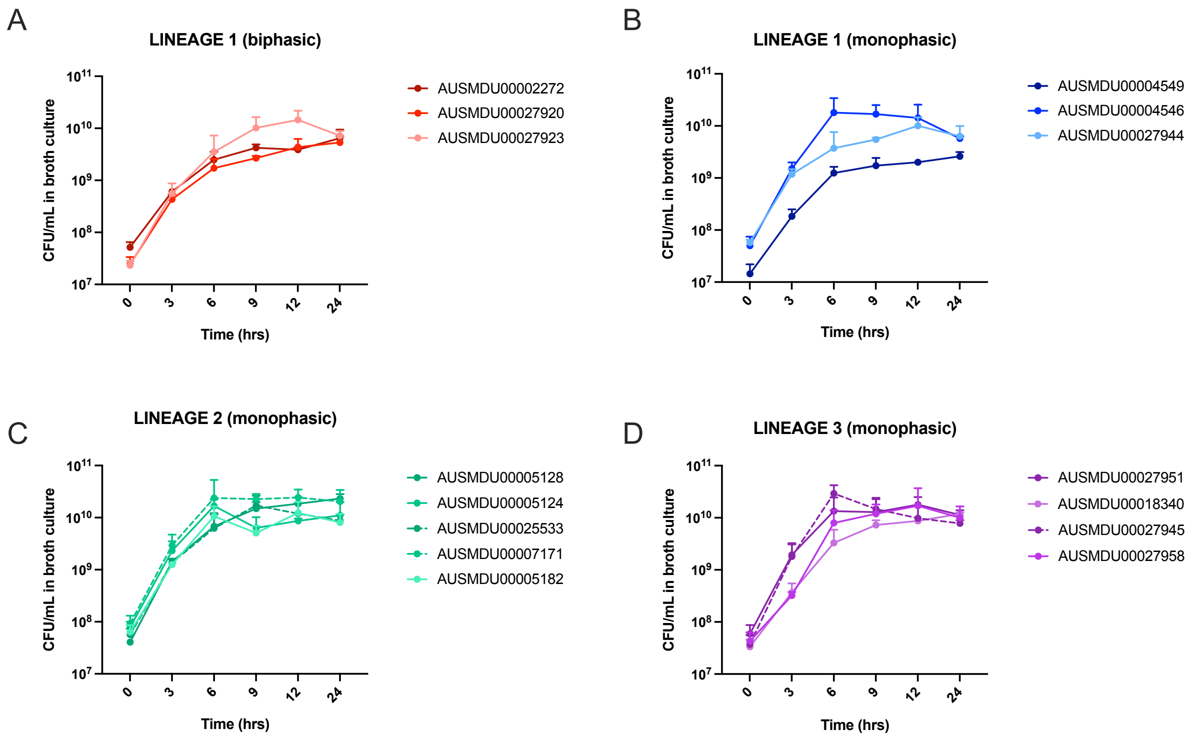

E

hpi	Lineage	Isolate comparison	Summary	Adjusted *p* value
0	1	AUSMDU00004549 vs. AUSMDU00027944	**	0.0054
3	1	AUSMDU00004549 vs. AUSMDU00027944	*	0.0432
9	1	AUSMDU00004549 vs. AUSMDU00027944	**	0.0055
24	1	AUSMDU00004549 vs. AUSMDU00004546	**	0.0069

Supplementary Figure 8: Growth of *Salmonella* 4,[5],12:i:- and ST34 biphasic lineage 1 *S.* Typhimurium isolates in nutrient broth.

Growth in nutrient broth over 24 hours **for A)** ST34 Lineage 1 biphasic *S.* Typhimurium, **B)** Lineage 1 *Salmonella* 4,[5],12:i:-, **C)** Lineage 2 *Salmonella* 4,[5],12:i:-, and **D)** Lineage 3 *Salmonella* 4,[5],12:i:- (D), as listed in Supplementary Table 1. Bacteria were plated onto Luria agar at selected timepoints (0, 3, 6, 9, 12, 24 hours) post initial broth inoculation and growth expressed as colony forming units CFU/mL of nutrient broth. Each point and centre of error bar represents the average CFU/mL of three independent biological replicates (n=3) and all error bars indicate +1 standard deviation from a 3 independent biological replicates. Each biological replicate was also performed in technical duplicate. **E)** Table of statistically significant differences between isolates within a lineage as determined by two-way ANOVA with Tukey's post-test for multiple comparisons. All other comparisons made between isolates

within each lineage were not significant. * = p<0.05, ** = p<0.01. CFU; colony forming units, hrs; hours.

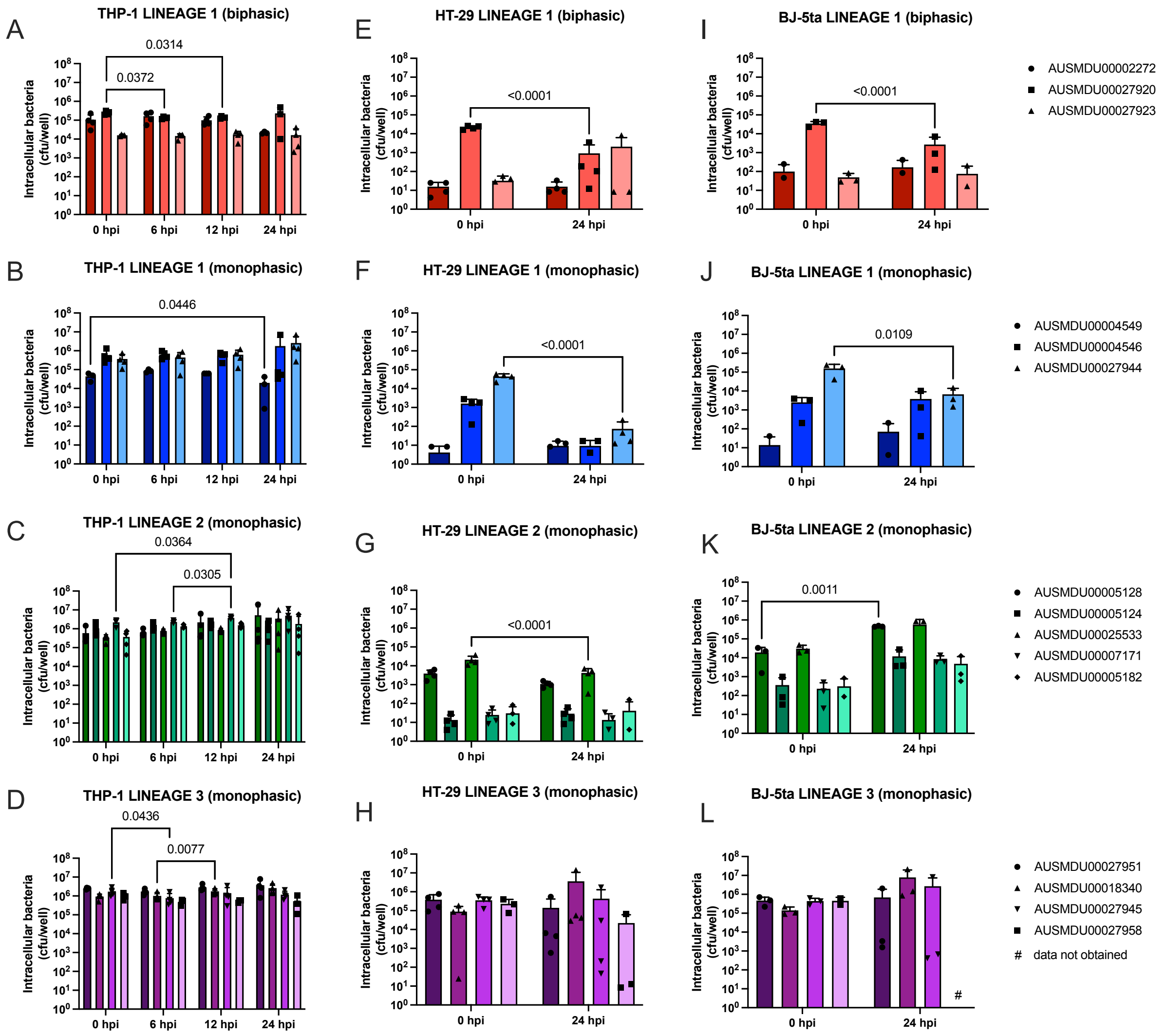

Supplementary Figure 9: Growth of *Salmonella* 4,[5],12:i:- and biphasic Lineage 1 *S.* Typhimurium isolates in THP-1, HT-29 and BJ-5ta cells.

Bacterial growth in **(A-D)** THP-1 cells, **(E-H)** HT-29 cells, and **(I-L)** BJ-5ta cells infected at MOI:10 with representative *Salmonella* 4,[5],12:i:- Lineage 1-3 isolates and ST34 Lineage 1 biphasic *S.* Typhimurium isolates (Supplementary Table 1). THP-1 cells were assayed at time 0, 6, 12 and 24 hpi. HT-29 and BJ-5ta were assayed at 0 and 24 hpi. Each point represents the CFU/well of 1 biological replicate, with the centre of the error bars indicating average of n=3-4 biological replicates + 1 standard deviation. Each biological replicate was also performed in technical duplicate. Statistical differences were determined by 2-way ANOVA with Tukey's post-test for multiple comparisons. MOI; multiplicity of infection, hpi; hours post-infection, CFU; colony forming units.

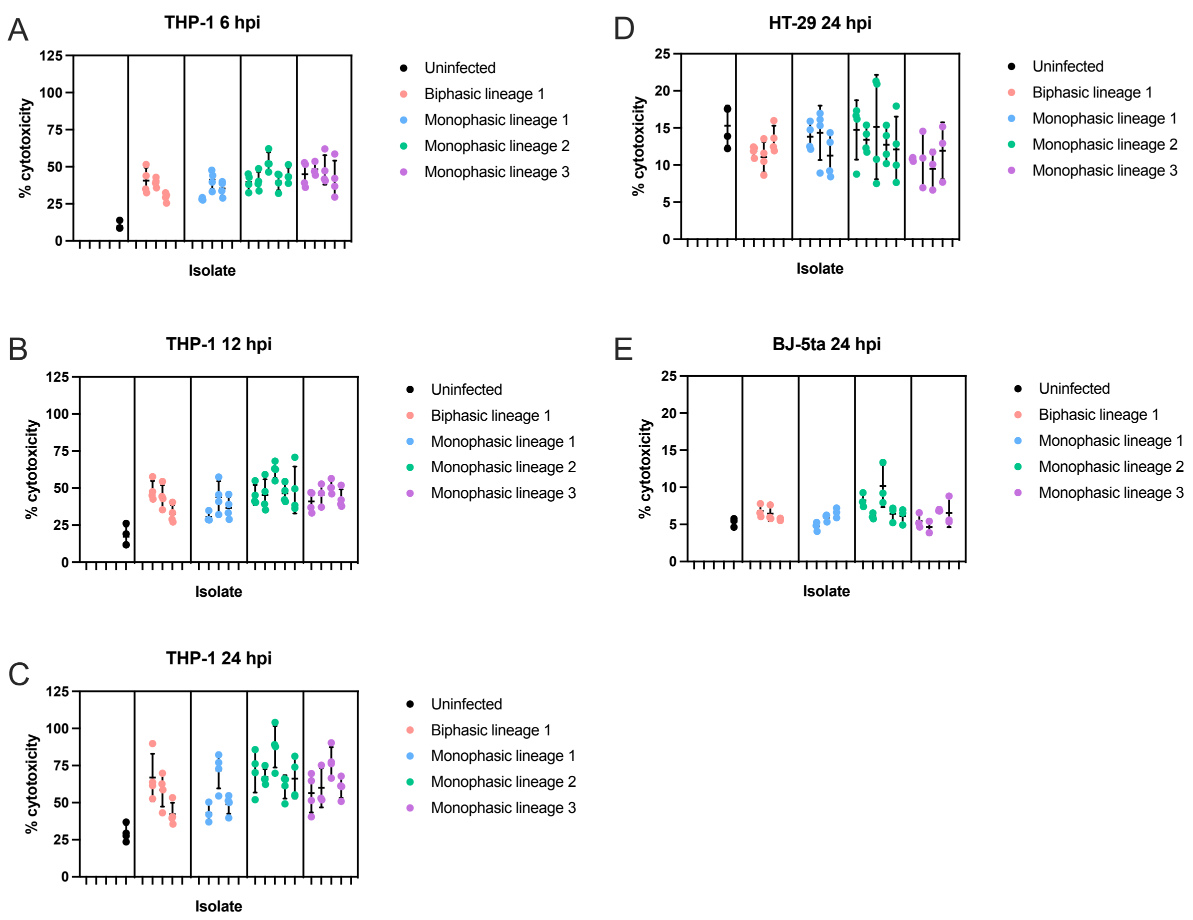

Supplementary Figure 10: Cytotoxicity in THP-1, HT-29 and BJ-5ta cells infected with *Salmonella* 4,[5],12:i:- and biphasic Lineage 1 *S.* Typhimurium isolates.

A-C) Differentiated THP-1 (human monocyte) cells, **(D)** HT-29 (human intestinal epithelial) cells, **(F)** or BJ-5ta (human fibroblast) cells were infected at MOI:10 with representative *Salmonella* 4,[5],12:i:- and ST34 Lineage 1 biphasic *S.* Typhimurium isolates (Supplementary Table 1). Cell supernatants from were assayed for LDH as a measure of cytopathic effects of infection, and % cytotoxicity calculated from total uninfected lysed control cells. Samples were collected from THP-1 cells at times 6, 12 and 24 hpi, and from HT-29 and BJ-5ta cells at 24 hpi. Each point represents the % cytotoxicity of 1 biological replicate, with the centre of the error bars indicating the average value of n=3-4 biological replicates + 1 standard deviation. Each biological replicate was also performed in technical duplicate. Statistical significance determined by nested one-way ANOVA with Tukey's multiple comparisons test. MOI; multiplicity of infection, hpi; hours post-infection, LDH; lactate dehydrogenase.

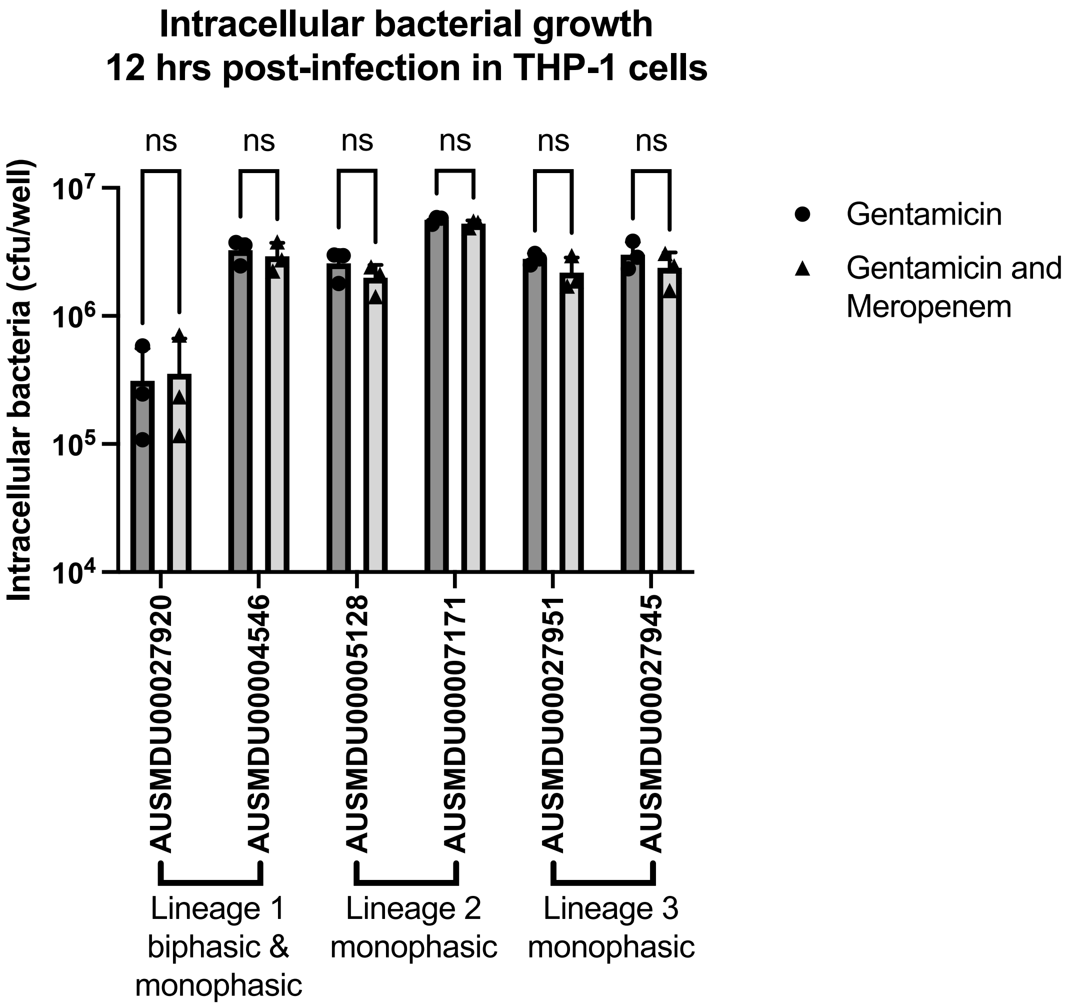

Supplementary Figure 11. The effect of meropenem (0.5µg/ml) in combination with gentamicin (10 µg/ml) in comparison to gentamicin alone (10 µg/ml) on intracellular bacterial growth of *Salmonella* in THP-1 cells 12 hours post-infection. Each point represents the CFU/well of 1 biological replicate, with the centre of the error bars indicating average of n=3 biological replicates + 1 standard deviation. Each biological replicate was also performed in technical duplicate. No significant difference in growth was observed across all lineages used in this study.

Supplementary Data 1: Data on the ST34 *Salmonella* 4,[5],12:i:- isolates included in this study

Supplementary Data 2: Details of Australian complete genomes.

Supplementary Table 1. List of isolates tested in phenotypic assays.

Isolate ID	Biphasic/ Monophasic	Lineage	Plasmids	Associated figure with genotypic data
AUSMDU00002272	Biphasic	Lineage 1	IncAC pST3	Fig 1, Supp Fig 3, 4
AUSMDU00027920	Biphasic	Lineage 1	IncAC pST3	Fig 1, Supp Fig 3, 4
AUSMDU00027923	Biphasic	Lineage 1	IncAC pST3	Fig 1, Supp Fig 3, 4
AUSMDU00004549	Monophasic	Lineage 1	IncAC pST3	Fig 1, 2B, 3B, Supp Fig 3, 4
AUSMDU00004546	Monophasic	Lineage 1	IncAC pST3	Fig 1, Supp Fig 3, 4
AUSMDU00027944	Monophasic	Lineage 1	IncAC pST3	Fig 1, 2B, 3B, Supp Fig 3, 4
AUSMDU00005128	Monophasic	Lineage 2	IncI pST23, IncHI2 pST4	Fig 1, Supp Fig 3, 4
AUSMDU00005124	Monophasic	Lineage 2	IncI pST23, IncHI2 pST4	Fig 1, 2C, Supp Fig 3, 4
AUSMDU00025533	Monophasic	Lineage 2	None	Fig 1, Supp Fig 3, 4
AUSMDU00007171	Monophasic	Lineage 2	None	Fig 1, 2C, Supp Fig 3, 4
AUSMDU00005182	Monophasic	Lineage 2	IncI pST23, IncHI2 pST4	Fig 1, 3B, Supp Fig 3, 4
AUSMDU00027951	Monophasic	Lineage 3	IncHI2 pST3	Fig 1, 2D, Supp Fig 3, 4
AUSMDU00018340	Monophasic	Lineage 3	IncHI2 pST3	Fig 1, 2D, 3B, Supp Fig 3, 4
AUSMDU00027945	Monophasic	Lineage 3	IncAC pST3	Fig 1, Supp Fig 3, 4
AUSMDU00027958	Monophasic	Lineage 3	IncHI2 pST3	Fig 1, Supp Fig 3, 4
