# Supplementary material for: Evolutionary dynamics of multidrug resistant Salmonella enterica serovar 4,[5],12:i:- in Australia
Source: Nat Commun. 2021 Aug 9;12:4786. doi: 10.1038/s41467-021-25073-w (PMC8352879; doi:10.1038/s41467-021-25073-w)
Supplement: Supplementary file 2 — Reporting Summary [file 41467_2021_25073_MOESM2_ESM.pdf]

nature portfolio

Corresponding author(s): Danielle J Ingle
Deborah A Williamson

Last updated by author(s): Jul 20, 2021

Reporting Summary

Nature Portfolio wishes to improve the reproducibility of the work that we publish. This form provides structure for consistency and transparency in reporting. For further information on Nature Portfolio policies, see our Editorial Policies and the Editorial Policy Checklist.

Statistics

For all statistical analyses, confirm that the following items are present in the figure legend, table legend, main text, or Methods section.

n/a	Confirmed	
☐	☒	The exact sample size (*n*) for each experimental group/condition, given as a discrete number and unit of measurement
☐	☒	A statement on whether measurements were taken from distinct samples or whether the same sample was measured repeatedly
☐	☒	The statistical test(s) used AND whether they are one- or two-sided<br>*Only common tests should be described solely by name; describe more complex techniques in the Methods section.*
☒	☐	A description of all covariates tested
☐	☒	A description of any assumptions or corrections, such as tests of normality and adjustment for multiple comparisons
☐	☒	A full description of the statistical parameters including central tendency (e.g. means) or other basic estimates (e.g. regression coefficient) AND variation (e.g. standard deviation) or associated estimates of uncertainty (e.g. confidence intervals)
☐	☒	For null hypothesis testing, the test statistic (e.g. *F*, *t*, *r*) with confidence intervals, effect sizes, degrees of freedom and *P* value noted<br>*Give P values as exact values whenever suitable.*
☐	☒	For Bayesian analysis, information on the choice of priors and Markov chain Monte Carlo settings
☒	☐	For hierarchical and complex designs, identification of the appropriate level for tests and full reporting of outcomes
☒	☐	Estimates of effect sizes (e.g. Cohen's *d*, Pearson's *r*), indicating how they were calculated

Our web collection on statistics for biologists contains articles on many of the points above.

Software and code

Policy information about availability of computer code

Data collection	No software was used for data collection
Data analysis	Core alignment was performed using Snippy v4.3.7, phaster, Gubbins and SNP-sites v2.5.1<br>Genomes were assembled with Spades v3.13<br>Phylogenetic analysis was performed with IQ-TREE v1.6.10<br>Antimicrobial resistance genes were detected using AbritAMR (https://github.com/MDU-PHL/abritamr) in conjunction with the AMRFinder database v3.2.1<br>In silico serovar SISTR v1<br>Phase II region Snippy v4.6.0 and Panaroo v1.2.4<br>Complete genome assemblies Unicycler v0.4.8-beta (hybrid) or HGAP3 - SMRTPortalv2.3.0, Porechop v0.2.4, Filtlong v0.2.0 and annotated with Prokka v1.14.0<br>Bayesian analysis with BEAST 1.10.4, TempEst v1.5, TreeAnnotator v1.10.4 and SkyGrowth (https://github.com/mrc-ide/skygrowth)<br>EMBOSS (v6.6.0).<br>Plasmid MLST https://pubmlst.org/plasmid/ and ARIBA v2.14.1<br>Heavy metals AMRFinderPlus v3.2.1 and ABRicate v0.9.7<br>Visualisation was performed in R v3.6.1 using packages vegan v2.5-6, GenoPlotR v0.8.9, pheatmap v1.0.12, ggplot2 v3.2.1, ggtree v1.16.6, circlize v0.4.8, ape v5.3 and tidyverse v1.2.1.<br>Phenotypic experiments analysed with Prism software (GraphPad Software v9.0)

For manuscripts utilizing custom algorithms or software that are central to the research but not yet described in published literature, software must be made available to editors and reviewers. We strongly encourage code deposition in a community repository (e.g. GitHub). See the Nature Portfolio guidelines for submitting code & software for further information.

Data

Policy information about availability of data

All manuscripts must include a data availability statement. This statement should provide the following information, where applicable:

- Accession codes, unique identifiers, or web links for publicly available datasets
- A description of any restrictions on data availability
- For clinical datasets or third party data, please ensure that the statement adheres to our policy

Supplementary Data 1 lists the individual accessions for all isolates, with associated metadata. Short read data for Australian isolates in this study are available from the NCBI Sequence Read Archive (BioProject PRJNA319593 https://www.ncbi.nlm.nih.gov/bioproject/319593 or PRJNA556438 https://www.ncbi.nlm.nih.gov/bioproject/556438). Long-read data are available at the European Nucleotide Archive (ENA) under PRJEB41036 https://www.ebi.ac.uk/ena/browser/view/PRJEB41036, and individual accessions are provided in Supplementary Data 2. An interactive annotated phylogeny is available in Microreact https://microreact.org/project/mfxxBchBsUpsJu7nvfkFw4. Antimicrobial resistance genes were detected using AbritAMR (https://github.com/MDU-PHL/abritamr) in conjunction with the AMRFinder database v3.2.1 (https://github.com/ncbi/amr). Source data for phenotypic work are provided with this paper. Data supporting the findings of this study are available within the text and in Supplementary files.

Field-specific reporting

Please select the one below that is the best fit for your research. If you are not sure, read the appropriate sections before making your selection.

☐ Life sciences ☐ Behavioural & social sciences ☒ Ecological, evolutionary & environmental sciences

For a reference copy of the document with all sections, see nature.com/documents/nr-reporting-summary-flat.pdf

Ecological, evolutionary & environmental sciences study design

All studies must disclose on these points even when the disclosure is negative.

Study description	Research study investigating the evolution and emergence of Salmonella 4,[5],12:i:- in Australia.
Research sample	The research sample included 309 isolates from the Microbiological Diagnostic Unit Public Health Laboratory MDU PHL collection between 2007 to 2017. To provide geographical context, published data of Salmonella 4,[5],12:i:- isolates typed as ST 34 were included in this study from publicly available datasets. These data were comprised of isolates from Australia (n = 2), Italy (n = 13), the United Kingdom (n = 65), the United States of America (n = 63) and Vietnam (n = 30) and represented data from previous phylogenetic studies of Salmonella 4,[5],12:i:- from Europe, North America and South East Asia. Full details (including region, year, published study and accession) of all genomes included can be found in Supplementary Data 1.
Sampling strategy	1. All Salmonella 4,[5],12:i:- isolates in the MDU PHL collection that were phenotypically resistant to third generation cephalosporins (3GCs) were included, along with a random sample of Salmonella 4,[5],12:i:- or S. Typhimurium isolates with the ASSuT resistance profile, over an eleven year time frame from 2007 to 2017. This was done to capture the 3GCs isolates and the diversity in the MDU PHL collection of isolates with the ASSuT profile.<br>2. International isolates were included from previously published studies to provide context if they were Salmonella 4,[5],12:i:- isolates typed as ST 34, had geographical and temporal data available and had short read data. A random subsample of isolates was taken from the Elknekave et al study to ensure balance between the public and Australian data.<br>3. Details of the strategy are described in the methods section 'Sampling strategy'. The final sample size was suitable Bayesian analyses of the Salmonella 4,[5],12:i:-.
Data collection	1. Details of the sources for all isolates are provided in Supplementary Data 1.<br>2. Data collection of the international contextual isolates from previous studies are available in the respective studies. The international contextual isolates were downloaded from the SRA or ENA by DJI. For data included in published studies, metadata (including geographic origin, year of isolation, etc) was extracted from the supplementary information of those studies and included in our master spreadsheet.<br>3. Growth in broth was done by RLA and JSP: The CFU/mL measurements were performed in duplicate for each experiment, and the experiment was repeated three times on different days. Samples were collected from the same duplicate wells over the time course of each experiment.<br>4. Growth in mammalian cells was done by RLA and JSP: For CFU/well and LDH supernatant samples, each isolate was tested in duplicate for each experiment. For each isolate, CFU/well and LDH were measured as technical replicates from the same well, with independent wells assayed over time (due to having to lyse the cells to collect a sample). This experiment was repeated 3-4 times on separate days.
Timing and spatial scale	The dataset encompasses Salmonella 4,[5],12:i:- collected between 2006 - 2017 from five different geographic regions (Oceania, South East Asia, South East Europe, North West Europe and the Americas.<br>1 The Australian data spans 2007-2017 with either reported travel to South East Asia or no reported travel (Oceania) and matches the timespan for the international contextual isolates.<br>2.The previously data spans 2006-2017 from key Salmonella 4,[5],12:i:- studies and captures the diversity of Salmonella 4,[5],12:i:- circulating globally.<br>Details of the year and source of isolates are available in Supplementary Data 1 and Supplementary Fig 1.

Data exclusions	International contextual isolates from previous studies were excluded if they were not Salmonella 4,[5],12:i:- isolates, were not typed as ST34, lacked geographical and year of collection data and didn't have short read data available. Additional isolates were excluded from Elknekave et al by a random selection of isolates to include so these data overwhelm the Australian data which was the focus of the study. Genomes were excluded if the average depth across the reference genome was <50 and if the number of contigs >200 (filtering contigs for <500bases).
Reproducibility	All data, tools and software are publicly available. Details of parameters are given in the Methods.
Randomization	Randomization was not applicable to this study as the research as isolates were included based on a sampling strategy with no predetermined groups defined before the study commenced.
Blinding	Blinding was not relevant to this study as it is not a clinical trial

Did the study involve field work? ☐ Yes ☒ No

Reporting for specific materials, systems and methods

We require information from authors about some types of materials, experimental systems and methods used in many studies. Here, indicate whether each material, system or method listed is relevant to your study. If you are not sure if a list item applies to your research, read the appropriate section before selecting a response.

Materials & experimental systems

n/a	Involved in the study
☒	☐ Antibodies
☐	☒ Eukaryotic cell lines
☒	☐ Palaeontology and archaeology
☒	☐ Animals and other organisms
☒	☐ Human research participants
☒	☐ Clinical data
☒	☐ Dual use research of concern

Methods

n/a	Involved in the study
☒	☐ ChIP-seq
☒	☐ Flow cytometry
☒	☐ MRI-based neuroimaging

Eukaryotic cell lines

Policy information about cell lines

Cell line source(s)	Human macrophage (THP-1) (ATCC® TIB-202™)<br>Colonic epithelial (HT-29) (ATCC® HTB-38™)<br>Human h-TERT immortalized foreskin (BJ-5ta) (ATCC® CRL-4001™)
Authentication	For each cell line, we performed ATCC human cell STR profiling upon receipt of the cell line and following every 20 passage of the cell line.
Mycoplasma contamination	All cell lines are routinely tested for Mycoplasma contamination and all cell lines tested negative during experiments conducted for this study.
Commonly misidentified lines (See ICLAC register)	*Name any commonly misidentified cell lines used in the study and provide a rationale for their use.*
