# Supplementary material for: Evolutionary dynamics of multidrug resistant Salmonella enterica serovar 4,[5],12:i:- in Australia
Source: Nat Commun. 2021 Aug 9;12:4786. doi: 10.1038/s41467-021-25073-w (PMC8352879; doi:10.1038/s41467-021-25073-w)
Supplement: Supplementary file 3 — Description of Additional Supplementary Files [file 41467_2021_25073_MOESM3_ESM.pdf]

Description of Additional Supplementary Files

Supplementary Data 1: Data on the ST34 *Salmonella* 4,[5],12:i:- isolates included in this study

Supplementary Data 2: Details of Australian complete genomes.
